# The directed migration of gonadal distal tip cells in *Caenorhabditis elegans* requires NGAT-1, a ß1,4-N-acetylgalactosaminyltransferase enzyme

**DOI:** 10.1371/journal.pone.0183049

**Published:** 2017-08-17

**Authors:** Joseph Veyhl, Robert J. Dunn, Wendy L. Johnston, Alexa Bennett, Lijia W. Zhang, James W. Dennis, Harry Schachter, Joseph G. Culotti

**Affiliations:** 1 Department of Molecular Genetics, University of Toronto, Toronto, Ontario, Canada; 2 Lunenfeld-Tanenbaum Research Institute of Mt. Sinai Hospital, Toronto, Ontario, Canada; 3 Centre for Research in Neuroscience, McGill University, Montreal, Quebec, Canada; 4 Laboratory Medicine and Pathobiology, University of Toronto, Toronto, Ontario Canada; 5 Department of Molecular Medicine, Hospital for Sick Children, Toronto, Ontario, Canada; East Carolina University, UNITED STATES

## Abstract

Glycoproteins such as growth factor receptors and extracellular matrix have well-known functions in development and cancer progression, however, the glycans at sites of modification are often heterogeneous molecular populations which makes their functional characterization challenging. Here we provide evidence for a specific, discrete, well-defined glycan modification and regulation of a stage-specific cell migration in *Caenorhabditis elegans*. We show that a chain-terminating, putative null mutation in the gene encoding a predicted β1,4-N-acetylgalactosaminyltransferase, named *ngat-1*, causes a maternally rescued temperature sensitive (ts) defect in the second phase of the three phase migration pattern of the posterior, but not the anterior, hermaphrodite Distal Tip Cell (DTC). An amino-terminal partial deletion of *ngat-1* causes a similar but lower penetrance ts phenotype. The existence of multiple ts alleles with distinctly different molecular DNA lesions, neither of which is likely to encode a ts protein, indicates that NGAT-1 normally prevents innate temperature sensitivity for phase 2 DTC pathfinding. Temperature shift analyses indicate that the ts period for the *ngat-1* mutant defect ends by the beginning of post-embryonic development–nearly 3 full larval stages prior to the defective phase 2 migration affected by *ngat-1* mutations. NGAT-1 homologs generate glycan-terminal GalNAc-β1-4GlcNAc, referred to as LacdiNAc modifications, on glycoproteins and glycolipids. We also found that the absence of the GnT1/Mgat1 activity [UDP-N-acetyl-D-glucosamine:α-3-D-mannoside β-1,2-N-acetylglucosaminyltransferase 1 (encoded by *C*. *elegans gly-12*, *gly-13*, and *gly-14* and homologous to vertebrate GnT1/Mgat1)], causes a similar spectrum of DTC phenotypes as *ngat-1* mutations–primarily affecting posterior DTC phase 2 migration and preventing manifestation of the same innate ts period as *ngat-1*. GnT1/Mgat1 is a medial Golgi enzyme known to modify mannose residues and initiate N-glycan branching, an essential step in the biosynthesis of hybrid, paucimannose and complex-type N-glycans. Quadruple mutant animals bearing putative null mutations in *ngat-1* and the three GnT genes (*gly-12*, *gly-13*, *gly-14*) were not enhanced for DTC migration defects, suggesting NGAT-1 and GnT1 act in the same pathway. These findings suggest that GnTI generates an N-glycan substrate for NGAT-1 modification, which is required at restrictive temperature (25°C) to prevent, stabilize, reverse or compensate a perinatal thermo-labile process (or structure) causing late larval stage DTC phase 2 migration errors.

## Introduction

**Fig 1:**
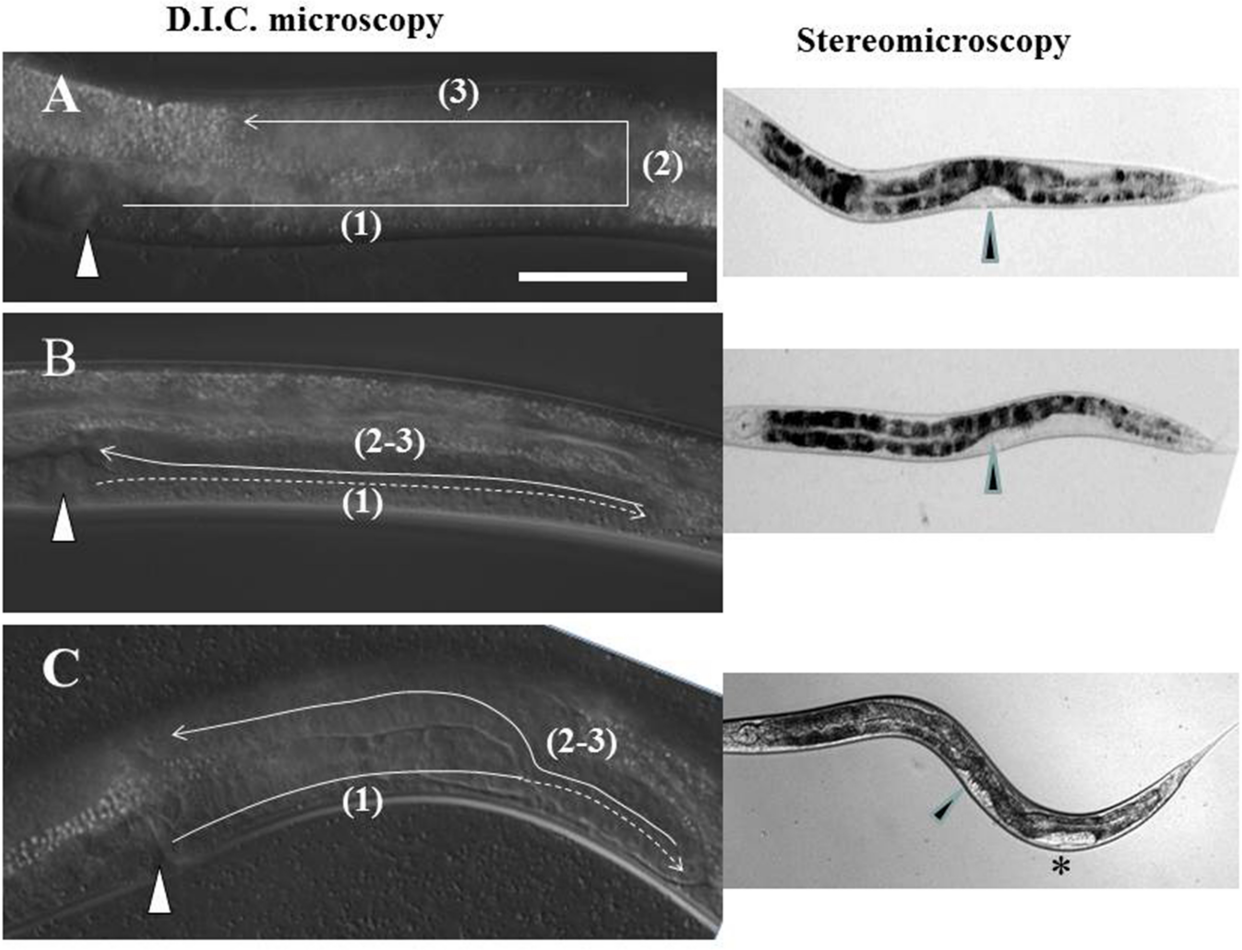
Common posterior Distal Tip Cell (DTC) trajectories in *ngat-1(ev821)* mutant animals. Lateral view of the posterior gonad arm in L4 hermaphrodite animals by D.I.C. microscopy. In all panels, anterior is left, dorsal is up. (**A**) The 2 DTCs are born in ventral mid-body (in position of future vulva marked by triangle) and normally migrate post-embryonically in 3 phases [(1) thru (3)] to shape the developing U-shaped gonad arms. (**B**) Some *ngat-1(ev821)* animals grown at 25°C have a normal posterior DTC migration as in panel **A**, while many have an *unc-6* mutant-like phase 2 failure with phases 2 and 3 executed on the ventral side (panel **B**), while others (panel **C**) show a partial return to mid-body before migrating dorsally. To the right of panels **B** and **C** are the predicted corresponding ventral clear patch (VCP) phenotypes visible by low magnification stereomicroscopy (asterisk marks partial VCP in panel **C**). Scale bar in panel **A** (for **A-C**) is 50 μm.

The molecular mechanisms that regulate and guide cell and axon migrations have relevance to a variety of human neurological and mental health disorders as well as metastatic cancer. In the past, we discovered that mutants of UNC-6/Netrin signaling affect both axon guidance and the migration of two cells in *C*. *elegans* called distal tip cells (DTCs). These cells migrate at the leading distal tip of each of the two independently extending hermaphrodite gonad arms to shape these arms during post-embryonic development [1]. The DTCs are born post-embryonically in the ventral mid-body and migrate in 3 phases (see **Fig 1A**): (1) longitudinally in opposite directions away from the mid-body—the anterior DTC along the right and the posterior DTC along the left ventral body wall muscles (BWMs), then (2) from ventral to dorsal BWMs across the lateral epidermis on the same side as the phase 1 migration, then (3) back to the mid-body along dorsal BWMs. The migratory path of the DTCs determines the final shape of the adult gonad arms, thus in the wild type the gonad arms are each U-shaped and mirror image when the trajectory is viewed from a lateral perspective. In the studies reported here, the DTC migration paths were deduced from the shape of the hermaphrodite gonad arms in 4^th^ larval stage (L4) animals and young adults (as in **Fig 1A**).

We previously showed that the secreted laminin-related guidance cue UNC-6/netrin requires the UNC-5 receptor acting alone or in combination with the UNC-40/DCC receptor to induce apparent repulsion from ventral sources of UNC-6 to guide ventral-to-dorsal (V-to-D) motor axon and phase 2 DTC migrations [1, 2]. In netrin signalling mutants (*unc-5*, *unc-6* and *unc-40* mutants) and the mutant subjects of this report, the phase 2 migration frequently fails to execute, nevertheless, the DTCs, stuck on the ventral side, move back to the mid-body along the ventral BWMs with proper phase 3 timing. This phase 2 migration failure causes a ventrally reflexed gonad arm in which the distal portion of the arm comes to lie beside the proximal portion of the same arm (as in **Fig 1B**). The side-by-side proximal and distal portions on the ventral side push the opaque intestine dorsally, creating a ventral clear patch (VCP) visible by low power stereo-microscopy in pre-adults (L4 stage larvae) and adults [see [3]]. Other kinds of DTC navigation errors cause smaller regions of gonad arms to lay side-by-side which manifest as smaller clear patches (as in **Fig 1C**). We crudely categorized VCPs as full (as in **Fig 1B**), near-full (full, but with a small opaque interruption), and partial (as in **Fig 1C**), depending on the number and extent of the clear patch(es) associated with each gonad arm. Clear patches therefore greatly facilitate scoring of a variety of DTC navigation errors.

The UNC-6/Netrin guidance system represents an important mechanism involving physical interaction and molecular cross-talk between DTCs and the extracellular matrix (ECM) during the course of migration. Further evidence for the importance of the ECM in DTC migration is that a variety of ECM molecules, their receptors, and enzymes that create or modify glycans present on ECM glycoproteins are known to affect DTC migration and in some cases also axon migration [4, 5]. These include proteoglycans UNC-52/perlecan [6] and SDN-1/syndecan [7], SQV-5/chondroitin synthase [8], HST-2/ heparan sulfate modifying enzyme [9], plus integrin related proteins PAT-4/ILK [10] and PAT-3/beta-integrin [11].

As development proceeds, the ECM is modified to form an acceptable substratum for the guidance of DTC and axon migrations. Genetic analysis demonstrated that two ADAMTS family matrix metalloproteases, GON-1 and MIG-17 are required to regulate DTC migration [12, 13]. GON-1, expressed in DTCs and BWMs, is essential for a normal rate of DTC migration while MIG-17, expressed in BWMs and localized to the gonad and DTC basement membrane, is essential for normal phases 2 and 3 DTC pathfinding. These results indicate that *mig-17*, like *unc-5*, *-6* and *-40*, is not required for DTC migration per se, but influences the navigation of phase 2 and phase 3 migrations—albeit in a different way than do the netrin signaling genes (see below).

It has been proposed that GON-1 and MIG-17 regulate DTC migration by remodeling the extracellular or basement membrane substrata used for migration [12, 13]. How metalloproteases like MIG-17 help to do this remains to be elucidated, however, secretion of MIG-17 from BWM and proper localization to the gonadal/DTC surface [12, 14] requires N-glycosylation of its pro-domain [14, 15] and on the basement membrane protein MIG-6/papilin [16]. Poorly glycosylated MIG-17, as predicted by its consequent mis-localization, causes defects in DTC migration [15]. Several genes required for normal glycosylation, including those encoding Golgi complex proteins COGC-1 to 8 [17] and MIG-23, a Golgi-resident NDPase [15, 17] are required to glycosylate MIG-17 and probably other basement membrane glycoproteins. Mutants of the above glycosylation genes have DTC defects resembling those of *mig-17* loss-of-function mutants in that the DTCs often meander widely along the dorso-ventral axis as they return to mid-body during phase 3 –a phenotype largely different from most of the phase 2 navigation errors of the netrin signaling mutants. Several papers have identified many additional genes required for DTC migration [18–24], but few are characterized beyond describing the DTC phenotypes induced by gene disruptions.

Proteins translated in the secretory pathway are N-glycosylated by covalent bonding of glycans to Asn (N) at NXS/T(X≠P) sites during translation and extrusion into the lumen of the endoplasmic reticulum (ER) [25]. The glycan portion of Glc_3_Man_9_GlcNAc_2_-pp-dolichol is transferred and serves as a ligand for chaperones calnexin and calreticulin, which bind the Glc-Man epitope and promote folding of the N-glycosylated polypeptide [26]. Glycoproteins traffic from the ER to the cis-Golgi where the N-glycans are trimmed by alpha-mannosidases and then rebuilt by the branching N-acetylglucosaminyltransferases (GnT1/Mgat1, Mgat2, Mgat4a,b,c and Mgat5). *C*. *elegans* has homologues for GnT1/Mgat1 (GLY-12, GLY-13, GLY-14), Mgat2 (GLY-20) and Mgat5 (GLY-2). In contrast to core glycan synthesis, which is constitutive in most cell types, the addition of branching and terminal sugars is often regulated in a tissue- or cell lineage–specific manner [27, 28]. Changes in terminal glycan structure are also often associated with malignant transformation in cancer [29–31] and metastasis [25, 29]. Tissue- and/or lineage-specific regulation of outer-chain biosynthesis is regulated by expression of the relevant glycosyltransferases, supply of the sugar-nucleotide substrates, and in some cases, by competition between enzymes for a common acceptor, however, the majority of regulated terminal glycosylations observed do not yet have a defined function.

Here we identify and characterize mutations in *C*. *elegans* genes encoding 2 types of glycosyltransferases that cause aberrant phase 2 DTC migration. One gene, *ngat-1*, that was identified by a mutation that causes VCPs, was cloned and found to encode a predicted β-1,4-N-acetylgalactosaminyl transferase predicted to generate a LacdiNAc glyco-epitope near or at the distal termini of oligosaccharides that decorate one or more unknown glycoproteins or glycolipids. The *ngat-1* mutant has an unusual DTC migration phenotype in that phase 2 migration failures are largely confined to the posterior DTC. The *ngat-1* mutants are also temperature sensitive (ts) for the posterior DTC migration defects with a ts period that ends just prior to or early during the first larval stage. The phase 2 navigation defects, largely confined to the posterior DTC, and the temperature-sensitivity of the *ngat-1* mutants are unusual phenotypes that we discovered are also shared with the triple null mutant of three genes (*gly-12*, *gly-13* and *gly-14*). These genes each encode an enzymatically active UDP-N-acetyl-D-glucosamine:α-3-D-mannoside β-1,2-N-acetylglucosaminyltransferase I [32] (also known as GnT1 or Mgat1), and together encode all of this Golgi enzyme activity, which is essential for hybrid, paucimannose, and complex-type N-glycan synthesis. The temperature-sensitive period for the *gly-14; gly -12 gly-13* triple *GnT1* mutant posterior DTC migration defects, as for the *ngat-1* mutant, ends by the first larval stage of development, which surprisingly is nearly three full larval stages prior to the phase 2 DTC migration affected by these mutations. This is an unusual finding for any gene known to regulate cell migration and supports the suggestion that NGAT-1 and GnT1 activities may modify the same N-glycoprotein target(s) required for normal phase 2 DTC migration—modifications that are required for robust adaptation in early development to temperature variation, as revealed by subsequent altered phase 2 DTC migration patterns.

## Materials and methods

### Nematode culture

Standard procedures were used for the culture, maintenance and genetic analysis of *C*. *elegans* [33, 34]. The N2 Bristol strain was used as the standard wild-type strain. The CB4856 Hawaiian strain was used for one-step SNP/whole genome sequencing (WGS). Mutants used in this study were: Linkage group X (LGX): *unc-6(ev400)* [1], *dpy-3(e27)*, *gly-12(ok712)*, *gly-13(id47)* [35], *dpy-3(e27)*, LG I: *bre-4(ok3167)*, *(dpy-5(e61)*, *unc-40(ev543)*, LG II: *dpy-10(e128)*, *ngat-1(ev821)*, *ngat-1(ev823)*, *unc-52(e444)*, LG III: *gly-14(id48)* [35], LG V: *him-5(e1490)* [36]. Males from two transgenic lines were used: CF702 *muIs32[mec-7*::*gfp; lin-15(+)]; him-5(e1490)*, and DE60 *dnIs13[gly-18p*::*gfp; unc-119(+)]; him-5(e1490)*.

Strains not isolated in our laboratories were obtained from the *Caenorhabditis elegans* Genetics Center, courtesy of T. Stiernagle (University of Minnesota).

### Genetic analyses

The *ngat-1(ev821)* and *ngat-1(ev823)* strains were backcrossed to N2 (wild type) at least two times before characterization. The *gly-14; gly-12 gly-13* triple mutant was originally isolated and made available by one of us (HS) for characterization. The mutant strains characterized in the Results were derived as follows. We crossed *ngat-1(ev821)* males to the *gly-14; gly-12 gly-13* triple to construct the *ngat-1; gly-14; gly-12 gly-13* quadruple mutant. In this process, we isolated *gly-14; gly-12 gly-13* triple and *ngat-1(ev821)* single mutant co-segregants of the quadruple, which should share, similar if not identical, genetic backgrounds with one another for purposes of comparison. For comparisons of penetrance for animals grown at the same temperature, the animals were grown in parallel, i.e., in the same incubator space to correct for variations in incubator temperature, which could account for somewhat different penetrance of defects observed following growth in the same incubator at different times. Differences in penetrance were also minimized by growing animals without starvation for at least two generations before scoring them for defects.

To separate *ev821* from *unc-40(ev543)*, we crossed double mutant hermaphrodites to males heterozygous for *dpy-5(e61)*, a closely linked marker used as a balancer for *unc-40(ev543)*, then cloned non-Unc F1s to identify clones segregating Dpy progeny. We failed to detect VCPs in the Dpy animals in the F2s, so we cloned 100 F2 Dpys and discovered several that produced F3s with significant penetrance of phase 2 DTC navigation errors–presumptive *dpy-5*; *ev821* double mutants. We then eliminated the *dpy-5* mutation by repeating the above procedure substituting N2 (wild type) males for *dpy-5* heterozygous males in the cross and selecting for VCPs in the F3 to derive *ev821* homozygotes lacking the *dpy-5* mutation (**Fig 1**).

The genetic characteristics of *ev821* were first assayed by crossing genetically marked males [*DE60 dnIs13[gly-18p*::*gfp; unc-119(+)]; him-5(e1490)* or *CF702 muIs32[mec-7*::*gfp; lin-15(+)]; him-5(e1490)*] to hermaphrodites homozygous for *ev821* and analyzing subsequent F1 cross- and F2, F3 and F4 self-fertilization progeny. From a related cross of *dpy-3* males to homozygous *ev821* hermaphrodites, we also analyzed subsequent F1 cross- and F2, F3 and F4 self-fertilization progeny with results similar to those obtained from the *CF702* and *DE60* crosses.

### WGS-SNP cloning of *ngat-1*

Mutant *ev821* (N2 Bristol background) homozygous males were crossed with CB4856 (Hawaiian background) hermaphrodites. F1s were cloned and grown at 20°C. Self-fertilization progeny of cloned F2s were assayed for worms that displayed the *ev821* ventral clear patch (VCP) phenotype. Forty-seven homozygous *ev821* F3 lines were grown by self-fertilization and DNA was made from each line. DNAs were pooled for WGS at 30x coverage. The Cloudmap program [37] was used to localize the genomic regions enriched for Bristol (non-Hawaiian) strain DNA. Bristol DNA was found to be greatly enriched relative to Hawaiian DNA in only one region of the genome, which is on LG II. This region contained 4 Bristol genes that appeared carry mutations in open-reading-frame (ORFs) relative to a standard N2 wild type strain (see Results).

### Molecular biology

Standard molecular biology methods [38] were used unless otherwise noted.

#### Transgenic constructs and germline transformation

The following constructs were newly designed and cloned in our lab. The sequences of plasmid constructs were verified by DNA sequence analysis.

#### *ngat-1* rescue genomic fragment

A 3297 bp PCR fragment containing the *ngat-1* gene was amplified from N2 genomic DNA. The fragment included 985 bps of sequence upstream of the initiator ATG and 633 bps of sequence downstream of the terminator TAA sequence.

#### *myo-3* promoter-driven *ngat-1* rescue fusion PCR (*myo-3p*::*ngat-1(+)*)

The *myo-3* promoter was amplified from pCFJ104 (*Pmyo-3*::*mCherry*::*unc-54*) (a gift from Erik Jorgensen) yielding a promoter fragment that included 2536 bp upstream of the initiator ATG sequence.

The *ngat-1* coding fragment of 2344 bps was amplified from fosmid WRM0637aH09 DNA, spanning from the initiator ATG to 698 bps downstream of the terminator TAA codon. These two fragments were joined using fusion PCR such that the initiator ATG of the *myo-3* gene substituted for the ATG of *ngat-1*.

#### *lag-2* promoter-driven *ngat-1* rescue fusion PCR (*lag-2p*::*ngat-1(+)*)

The *lag-2* promoter was amplified from pJK590 (gift from Judith Kimble) yielding a promoter fragment that included 3131 bps of *lag-2* promoter DNA upstream of the initiator ATG. The *ngat-1* coding sequence of 2344 bps was amplified from fosmid WRM0637aH09 DNA, spanning from the initiator ATG to 698 bps downstream of the terminator TAA codon. These two fragments were joined using fusion PCR such that the initiator ATG of the *lag-2* gene substituted for the ATG of *ngat-1*.

#### *ngat-1* promoter-driven GFP expression plasmid (*ngat-1p*::*gfp*)

Briefly, a 3181 bp *ngat-1* genomic sequence including 1592 bps of DNA upstream of the initiator ATG was amplified from N2 genomic DNA and inserted into plasmid pCRII-TOPO (Invitrogen). The *gfp* sequence was amplified from plasmid pPD95.70 (Addgene plasmid # 1492, a gift from A. Fire) that included the SV-40 nuclear localization signal at the N-terminus and the 3’ untranslated sequence of *unc-54*. The *gfp* sequence was inserted into *ngat-1* genomic sequence such that *ngat-1* initiator ATG codon replaced the initiator ATG of the *gfp* gene to create construct *ngat-1p*::*gfp* used for transgenic analysis of *ngat-1* gene expression.

#### *dpy-7* promoter-driven *ngat-1(+)* rescue construct (*dpy-7p*::*ngat-1(+)*)

The *dpy-7* promoter was amplified from N2 genomic DNA yielding a promoter fragment that included 369 bps upstream of the initiator ATG sequence. The *ngat-1* coding sequence of 2344 bps was amplified from fosmid WRM0637aH09 DNA, spanning from the initiator ATG to 698 bps downstream of the terminator TAA codon. These two fragments were joined using fusion PCR such that the initiator ATG of the *dpy-7* gene substituted for the ATG of *ngat-1*.

#### *elt-2* promoter-driven *ngat-1(+)* rescue construct (*elt-2p*::*ngat-1(+)*)

A 2310 bp genomic segment of *ngat-1* DNA sequence, that includes all six exons and the 3’ UTR, was inserted into plasmid pJM559 (58) downstream of the *elt-2* intestine-specific promoter sequence (5049 bps upstream of ATG) using Gibson assembly methods. In this construct, the initiator ATG of the *elt-*2 gene replaces the initiator ATG of the *ngat-1* gene

#### *dpy-7p*::*mCherry* epidermal reporter

To construct the *dpy-7* promoter-driven epidermal reporter, *mCherry* DNA was amplified by PCR and sub-cloned into pPD96.41 *dpy-7p*::*venus* (gift from A. Fire), using AgeI and EcoRI sites.

#### *odr-1p*::*reporter* co-transformation reporter

In some cases an *odr-1p*::*reporter* DNA [39] was co-injected as a co-transformation reporter.

#### *myo-2p*::*mCherry* co-transformation reporter

In some cases addgene plasmid # 19328 was co-injected as a co-transformation reporter.

### Germline transformation

Transgenic strains were generated by co-microinjection of the DNA mix into the distal gonad arms of hermaphrodites [40]. DNA mixes consisted of a test construct, a co-injection reporter, and carrier DNA to create a final DNA concentration of 100–150 ng/μl.

See **S3 Table** for details of DNA concentrations used for transgene injections.

#### Creation of a *ngat-1*::*gfp* expression strain

To examine *ngat-1* expression, we injected a solution containing the following DNAs: 100 ng/μl *ngat-1p*::*gfp* [pTG2-#7], 50 ng/μl *odr-1p*::*dsRed* (as co-transformation marker) into wild-type strain N2 (WT). The extrachromosomal array formed by this injection was integrated into the genome by irradiation to create *evIs460B[ngat-1p*::*gfp*, *odr-1p*::*ds red]*. We next injected 60 ng/μl *dpy-7p*::*mCherry* as epidermal reporter, and 40 ng/μl *PBS-KS(-)* into the strain carrying evIs460B to create *evEx481*, which was then used for fluorescence microscopic examination of *ngat-1* promoter driven expression of *gfp* relative to the epidermal *dpy-7* promoter-driven *mCherry*.

#### CRISPR Cas9 gene editing

A CRISPR guide RNA was designed to target the sequence TAGCATTACATAGATTAGTG within the first exon of the *ngat-1* gene. Plasmid pDD162 [41] expressing Cas9, the guide RNA and a visible co-injection marker were co-injected into the gonads of N2 worms and lines expressing the marker were recovered. Deletions and insertions in exon 1 were identified by polyacrylamide electrophoresis of PCR products and DNA sequence analysis.

### Temperature shift experiments

To determine the *ngat-1(ev821)* mutant temperature-sensitive (ts) period, eggs were collected from mutant adults grown at 16°C or 25°C, and placed on 4 plates which were then incubated at 16°C or 25°C, respectively. One plate was grown continuously at 16°C or 25°C, whereas three plates were shifted to 25°C or 16°C, respectively, at different stages of the animal's development–one during the L1 stage, one during the L2 stage and one during the L3 stage—then grown to a mix of L4 and young adult stages when they were assayed by D.I.C. for their DTC migration phenotypes.

To determine the *GnT1* triple knockout strain ts period, L1s were collected by settling in water from adults grown at 16°C or 25°C, and split into 2 portions for further growth—one portion was grown at the same temperature as the parents and the other shifted to the higher or lower temperature for subsequent growth to the L4 stage when they were scored for VCPs.

### Statistical analysis

Ninety-five percent upper confidence limits in error bars are for a binomial distribution of the same sample size and the observed proportion as mean for DTC phenotypes. Statistical tests were carried out using a standard (one-tailed) comparison of two proportions (Moore and McCabe, 1998). All p values represent the probability that the measured penetrance of the phenotype is significantly different between two strains. A p value less than 0.05 is considered to be significant. All comparisons described as significant in the Results were based on this criterion.

### Anatomical characterization

The *ngat-1(ev821)* mutant phenotype, a clear patch on the ventral side of the worm detected by low magnification stereo-microscopy, occurs as the result of DTC migration defects that affect the second phase of migration. The DTC defects were also assayed by D.I.C. microscopy to observe gonad arms of L4 stage and young adult animals. These stages were chosen because DTC migration is completed in the L4 stage and zygotes are not present in the uterus (even in young adults) to distort the shape of the gonad arms.

Worms were placed on a 2% agarose pad and immobilized with 5–10 μL of 25 μM sodium azide. The samples were examined using a Leica DMRA2 microscope set to DIC microscopy. Images were captured using a Hamamatsu ORCA-ER digital camera mounted to the microscope.

#### Microscopy

Strains that carried a *gfp* reporter were viewed using a Leica DMRXA microscope. In some cases, confocal microscopy was performed using a Leica DMFLS laser confocal microscope equipped with a 63 PC APO CS lens (1.40–0.60) or with a Nikon CSi laser confocal microscope. Confocal images were analyzed by processing confocal z-axis series using Volocity (Quorum Technologies) or ImageJ software (NCBI).

## Results

### *ev821* isolation and phenotypic characterization

Using a low-magnification stereo-microscope to screen for ventral clear patch (VCP) enhancers of DTC defects of a weak hypomorphic *unc-40(ev543)* allele, we discovered several mutations, one of which, *ev821*, we have characterized genetically and phenotypically. We first backcrossed the enhanced double mutant to isolate *ev821* homozygotes separated from *unc-40(ev543)* and propagated the *ev821* homozygotes (more readily identified in the F3 generation—see below) by several generations of self-fertilization. These homozygotes produced a substantial frequency of self-fertilization progeny with ventral clear patches (VCPs) of *unc-6* mutant-like phase 2 DTC migration failures (as in **Fig 1B**). As self-fertilizing hermaphrodites grown in parallel at room temperature (approximately 23°C) for several generations, the penetrance of the full plus near-full VCP phenotype (observed for posterior DTCs only and not including partial VCPs) in the *unc-40(ev543)* single mutant, the *unc-40(ev543); ev821* double mutant and *ev821* single mutant were 23% (n = 244), 55% (n = 268) and 38% (n = 418), respectively.

The penetrance of posterior VCPs in *ev821* single mutants grown at 25°C was somewhat higher than for mutant animals grown at room temperature and generally of 2 major types that occurred almost exclusively in the posterior gonad arm **(Fig 1)**. Thirty-nine percent (n = 115) of mutant animals had full or near-full VCPs in which the ventral clear patch extended from the vulva posteriorly to two-thirds of the distance from the vulva to the rectum (**Fig 1B**), and nearly as many (35%) had a partial clear patch (**Fig 1C**) that started some distance posterior to the vulva and extended a short distance toward the rectum.

**Fig 2:**
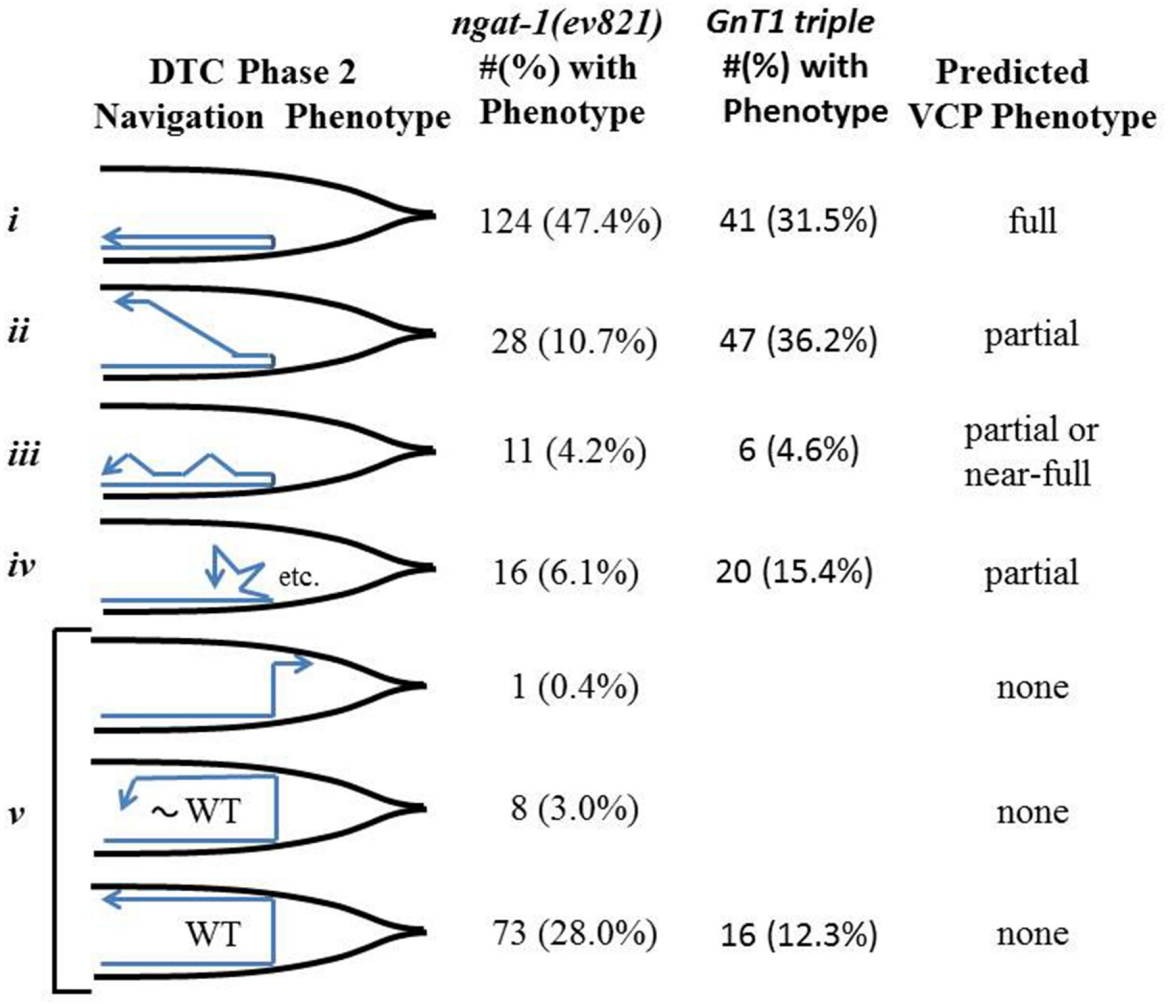
DTC navigational and ventral clear patch (VCP) phenotypes of *ngat-1(ev821)* animals. The number (#) and percentage (%) of *ngat-1(ev821)* and *GnT1* triple (see below) animals grown at 25°C with a given diagrammed posterior DTC abnormal (***i-iv***) or wild-type-like (WT = ***vi***) trajectory (deduced from posterior gonad arm shapes) are shown. Diagrams are based on D.I.C. microscopy of gonad arms from a lateral perspective. Predicted corresponding ventral clear patch (VCP) phenotypes are indicated in the right-most column.

When gonad arms of *ev821* mutant animals grown at 25°C were analyzed by higher magnification differential interference contrast (D.I.C.) microscopy, we found that 47% (n = 261) had a phase 2 DTC migration failure consistent with a full or near-full VCP (as in **Fig 1B** and **Fig 2 *i***), and that 25% had a morphology consistent with a partial clear patch (**Fig 2 *ii-iv***). Those consistent with a partial or near-full clear patch included animals in which the phase 2–3 migration started toward mid-body by migrating along the ventral BWMs, then migrated on a diagonal path to the dorsal BWMs (10.7%) (**Fig 2 *ii***), meandered ventrally along the dorso-ventral axis back toward mid-body (4.2%) (**Fig 2 *iii***), or changed direction frequently making multiple short excursions between turns (6.1%) (**Fig 2 *iv***). It is important to note that some of the animals scored as *unc-6* mutant-like phase 2 failures by D.I.C. microscopy sometimes had a DTC that made a dorsal excursion near the end of their phase 3 migration along the ventral side that might have appeared as partial VCPs by low power stereo- microscopy–this could readily account for the difference between the scores observed using stereo-microscopy compared with D.I.C. microscopy. Wild-type N2 animals grown at 25°C were not totally devoid of phase 2 DTC migration errors as 6 of 211 animals had phase 2 migration errors.

Nearly all of the observed phase 2 DTC migration errors occurred in the posterior gonad arm; the anterior arm was largely normal with fewer than 10% of animals showing anterior gonad arms with various abnormal morphologies, few of which resembled the phase 2 failures of the posterior DTC. In addition to the phase 2 posterior DTC migration errors, there were also a variety of DTC defects observed in the *ev821* mutant grown at 25°C, all of which were also observed to roughly the same extent in N2 wild-type animals grown at 25°C. There were frequent defects in phase 1 and phase 3 navigation. Anterior and posterior DTC phase 1 navigation defects were largely limited to gonad arms that migrated prematurely to the dorsal side during phase 1 (5 of 211 posterior DTCs and 5 of 199 anterior DTCs). Phase 3-specific navigation defects included distal gonad arms that returned to the ventral side following a normal phase 2 (see **Fig 2*v***), reversed direction on the dorsal side (2 of 199 anterior DTCs), or much more frequently migrated to the opposite side of the animal (switched sides) before stopping or continuing to migrate toward mid-body on the dorsal side. In rarer cases an entire gonad arm was found on the wrong side of the animal (e.g., 3 of 211 and 1 of 69 posterior DTCs in N2 and *ev821* animals, respectively). Many of the 25°C-generated anterior and posterior gonad arms, including ones exhibiting normal navigation, had a grossly enlarged phase 3-generated distal portion—fewer had a grossly enlarged phase 1-generated proximal portion. All of the above phases 1 and 3 phenotypes were also observed with roughly equal ferequencies in N2 wild-type animals grown at 25°C, suggesting they do not result from the *ngat-1* mutation, rather from growth at 25°C.

### Genetic characterization of *ngat-1(ev821)*

The genetic characteristics of *ev821* were first assayed by crossing genetically marked males (see Materials and methods) to hermaphrodites homozygous for *ev821*. The F1 cross progeny and subsequent generations of self-fertilization progeny were grown at 20°C. None of more than 1,000 F1s and only approximately 0.5% of the F2 animals from each cross had a full or near-full (not including partial) VCP phenotype (n = 2,071), compared to 29% for the starting *ev821* parent strain propagated for several generations at 20°C, suggesting the possibility of maternal rescue for the *ev821* gene (see below). From two of the above crosses, we cloned a total of 158 F2s and found that 36 of them (23%) segregated F3s with obvious VCPs, which is roughly the expected Mendelian frequency (25%) of *ev821* homozygotes among the F2s. From 3 additional reciprocal crosses of *ev821* males to genetically marked hermaphrodites, no F1s (0/200) or their self-fertilization F2 progeny (0/178) exhibited VCPs. From 178 cloned wild-type F2s, 50 (28%) segregated F3s with visible VCPs.

The relative absence of the VCP phenotype in the F1 and F2 generations and its subsequent reappearance in the F3 from the above crosses suggests a maternal rescue of the *ev821* gene phase 2 DTC migration defect. This was evident when the *ev821* allele in the F1came from the hermaphrodite or the male parent. These results, when considered together, suggest that *ev821* can be largely or totally rescued by a maternally or a zygotically functioning wild-type gene.

### Evidence for temperature-sensitivity in *ev821*

In order to determine if different growth temperatures affect the penetrance of VCPs or other aspects of the *ev821* phenotype, *ev821* worms were grown for a minimum of two generations without starvation at 16°C, 20°C, and 25°C. We observed that *ev821* animals grown at 16°C, 20°C and 25°C exhibited 9% (n = 200), 51% (n = 100) and 74% (n = 100) penetrance, respectively, for all 3 of the VCP phenotypes (including full, near-full, and partial VCPs) (Panel A in **S1 Fig**).

These results prompted us to repeat the first *ev821* inheritance experiment to determine if the maternal-effect is still apparent when *ev821* animals are crossed and grown at 25°C. In this experiment, none of 300 F1 worms had DTC defects, however, 14 of 15 cloned F1s segregated DTC migration defective (full or near-full VCP) F2 animals, which represented 2% (n = 1254) of the F2s—significantly higher (p<0.001 by one-tail z-test for difference between 2 proportions) than the 0.5% penetrance (n = 2071) among the F2 progeny grown at 20°C. Furthermore, 20 of the F2s with phase 2 DTC migration errors were cloned and all segregated substantial numbers of DTC defective VCP F3s. This shows that the degree of maternal rescue is reduced as the defect among the F2s becomes more penetrant at the higher temperature.

### *ev821* has a temperature-sensitive period for phase 2 DTC migration that ends near or soon after completion of embryogenesis

**Fig 3:**
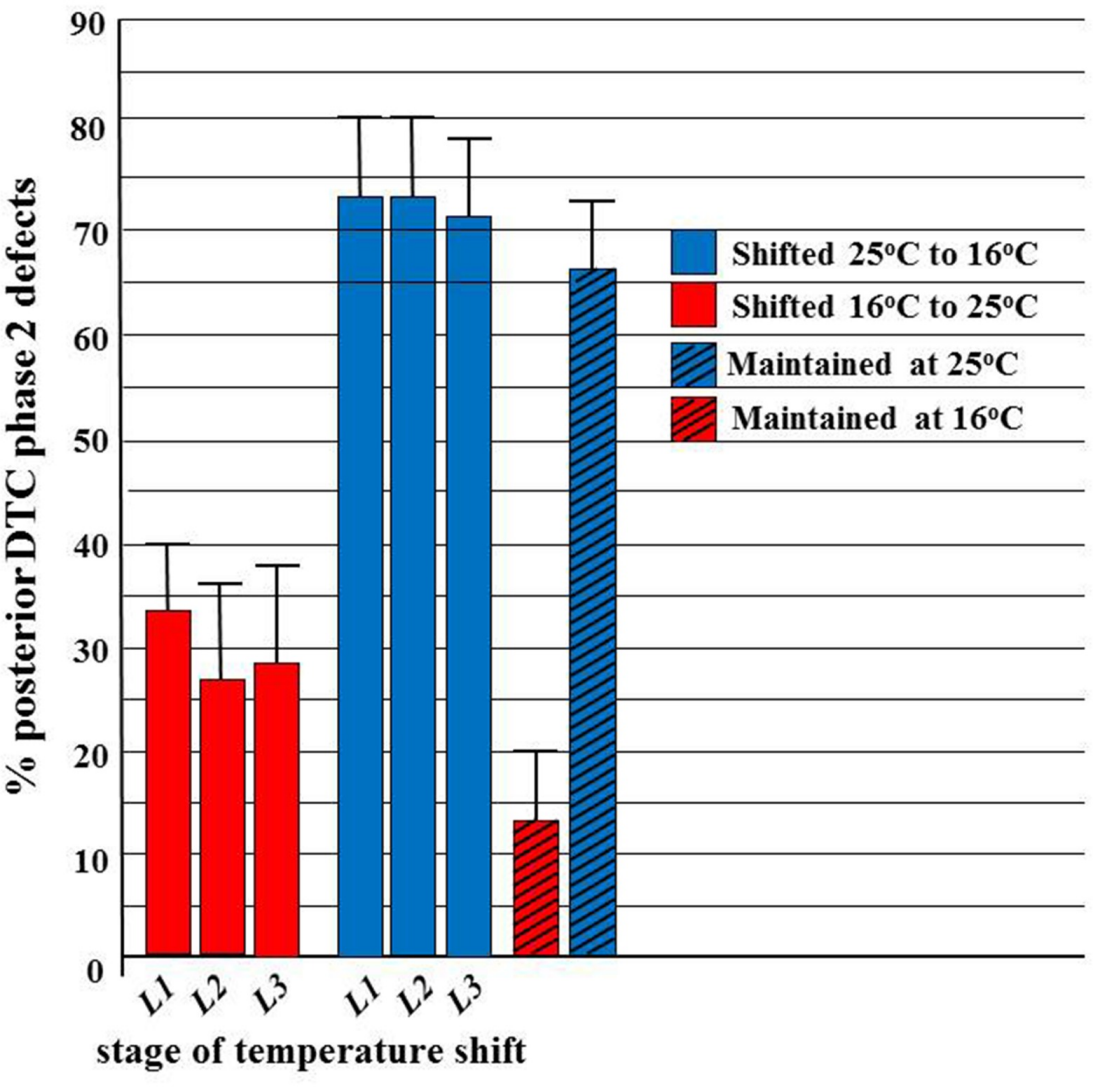
Approximate ts period for the *ev821* phase 2 DTC navigation errors. *ngat-1(ev821)* animals were grown at 16°C or 25°C then shifted to test temperature (25°C or 16°C, respectively) at various larval stages of development (L1, L2, and L3). Control animals were maintained at 16°C or 25°C test temperature throughout the experiment. Animals were scored by D.I.C. microscopy in the 4th larval stage (L4) or as young adults for phase 2 posterior DTC migration errors. A temperature-sensitive period was readily identified for these defects, which was developmentally much earlier than manifestation of the late L3 stage phase 2 DTC migration defect. Error bars represent z-test based 95% confidence intervals.

We also determined an approximate ts period for the *ev821* phase 2 DTC migration errors (detected using D.I.C. microscopy) by growing the animals at 16°C or 25°C for several generations then shifting to 25°C or 16°C, respectively, at various stages of development (**Fig 3**). The results shown in **Fig 3**demonstrate that embryonic growth at 25°C causes a high penetrance of phase 2 posterior DTC guidance defects (categorized in **S2 Fig**) approximately equal to the penetrance of defects that occurs if shifted back to 16°C during or after the first larval (L1) phase. This means that growth during the ts period (late embryogenesis) at 25°C is almost completely causal for the increased penetrance of these defects relative to growth at 16°C in the *ngat-1(ev821)* mutant. In contrast, *ngat-1(ev821)* mutant animals grown continuously at 16°C (including throughout the ts period) have a lower penetrance of phase 2 DTC defects, but this penetrance is slightly increased (from 13% to approximately 27%) by shifting to growth at 25°C during one of the L1 to L3 larval stages. The finding that some increased penetrance of the mutant phenotype can occur following a shift to growth at 25°C during early (e.g., L1) and even later larval stages suggests that growth at 16°C during the ts period does not entirely protect against mutant phase 2 DTC migration defects following a shift to 25°C post-embryonically, whereas, growth at 25°C during the ts period is nearly completely causal for the increased penetrance of these defects relative to growth at 16°C.

It is easy to lose sight of the fact that *ngat-1(+)* function in an animal does not just prevent the temperature sensitivity of the phase 2 DTC migration, but it also prevents the phase 2 DTC migration defect that occurs following growth at the permissive temperature. This realization raises the possibility that NGAT-1 does not necessarily directly, or even indirectly, affect the ts process (or structure) *per se* to prevent its thermo-lability and subsequent effect on DTC phase 2 migration, but rather may act by a completely different, possibly temperature-independent mechanism to somehow bypass or compensate for the innate ts process (or structure). Further experiments to determine the migration-relevant glycoprotein target(s) of NGAT-1 and GnT1 activities should help distinguish between these possibilities.

It is worth noting that mutants of *gale-1* which encodes UDP-glactosamine-4-epimerase, (interconverts UDP-galactose and UDP-glucose and in some animals UDP-N-acetylgalactosamine and UDP-N-acetylglucosamine), as well as *sqv-7*, which encodes a transporter of UDP- glucuronic acid, UDP-galactose, and UDP-N-acetylgalactosamine into the Golgi—also exhibit DTC migration defects that have not been extensively characterized [42]. Also of interest, an L1 period sensitive to rapidly alternating temperature changes, but not sensitive to a constant temperature, has been observed in miRNA *mir-34* and *mir-83* mutants at the time of DTC birth, but these mutations affect phases 1 and 3 of DTC migration [43]. The relationship of this form of temperature sensitivity to the temperature sensitivity of *ngat-1* and other mutants (see below) for phase 2 posterior DTC migration remains to be determined.

### Mapping and cloning of the *ngat-1* gene identified by the *ev821* mutation

The *ev821* mutation was identified by one-step SNP mapping/whole genome sequencing (WGS) [44]. SNP-WGS mapping of the *ev821* cross to CB4856 Hawaiian strain placed the *ev821* mutation within a chromosome II region between 11 and 11.5 Mb corresponding to around 3.5 cM (*sqt-1* is at 11.3 megabases at 3.44 cM). Comparison of the DNA sequence of the *ev821* and wild-type strains in this region indicated potential missense or predicted chain terminating nonsense mutations in four genes: *ptp-3/* protein tyrosine phosphatase, *rsf-1/*ras association domain protein, *D1043*.*1/* transcription regulatory protein, and *W02B12*.*11*/glycosyltransferase enzyme. To identify the *ev821* gene among these candidates, we carried out phenotypic rescue studies of the *ev821* mutant animals grown at 25°C, starting with *W02B12*.*11* (**S3 Fig**). Fosmid WRM0637aH09 (30 kB) spanning the *W02B12*.*11* gene significantly rescued the *ev821* DTC phenotype. A small (4.3kB) fragment of genomic DNA within this fosmid that included the *W02B12*.*11* gene, 0.9 kB of sequence upstream of the predicted transcription start site, plus 1.3 kB of sequence downstream of the predicted transcription termination site was amplified and found to also efficiently rescue the *ev821* posterior DTC phenotype—reducing the migration defects from 53% to 11% and strongly supporting the identification of *W02B12*.*11* as responsible for the *ev821* mutant DTC migration defect.

**Fig 4:**
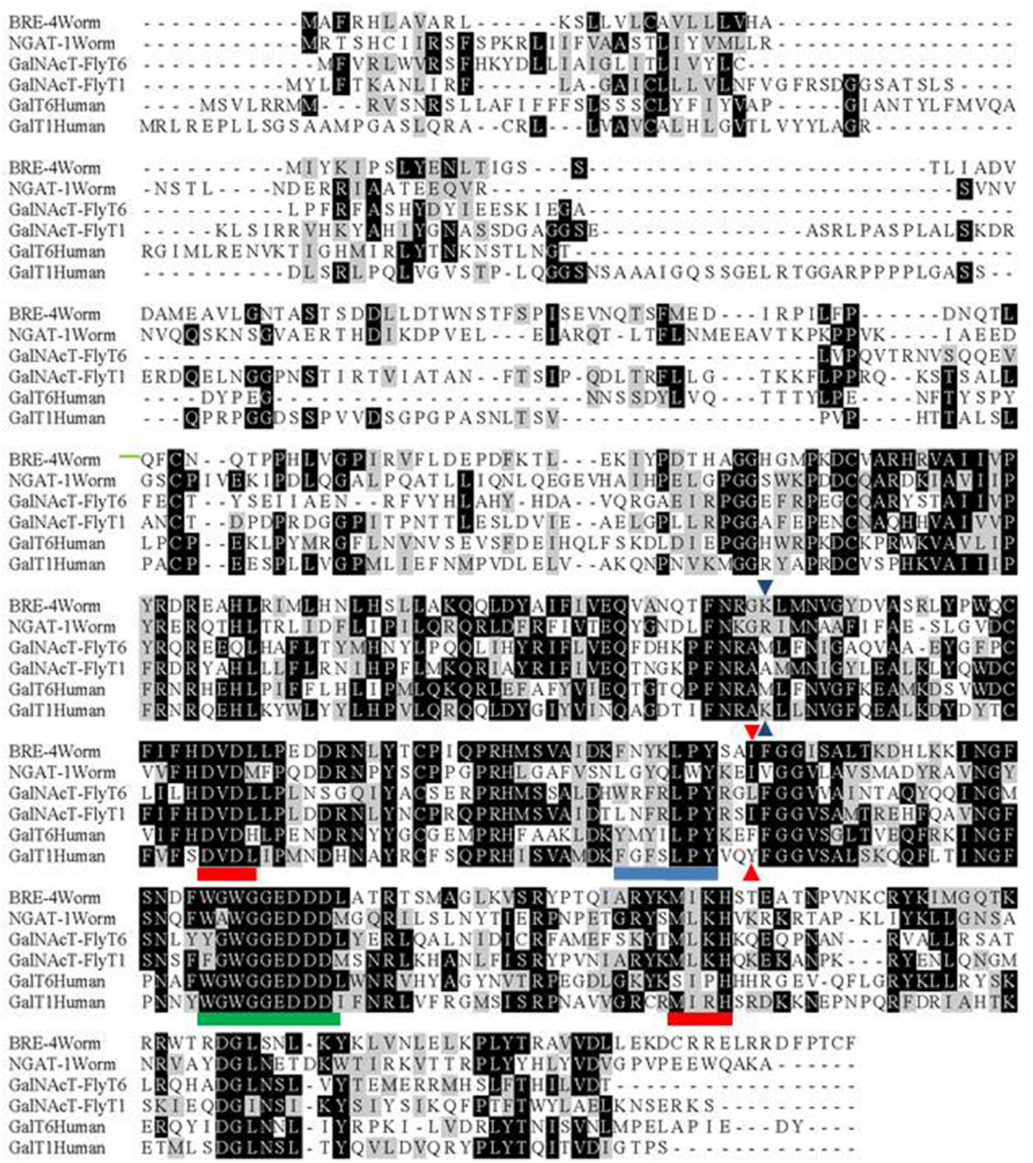
Comparison of galactosyl transferases from *C*. *elegans*, *D*. *melanogaster* (fly), and humans. Alignments were made of the most closely-related *C*. *elegans* (wormbase), fly (FBgn0027538, FBgn0031495) and human (Q8NCL4, Q10472) galactosyltransferases using CLUSTAL [47]. The *ngat-1(ev821)* nonsense mutation is indicated by blue triangles and the position of the Leu (or in this case Ile) residue, which specifies N-acetylgalactosamine transfer (vs Tyr or Phe, which do not) [48] is indicated by red triangles. Metal ion (red underline), acceptor (blue underline), and donor (green underline) binding sites as predicted from human galactosyl transferases [45]. Identical residues are shaded black and similar residues are shaded grey.

**Fig 5:**
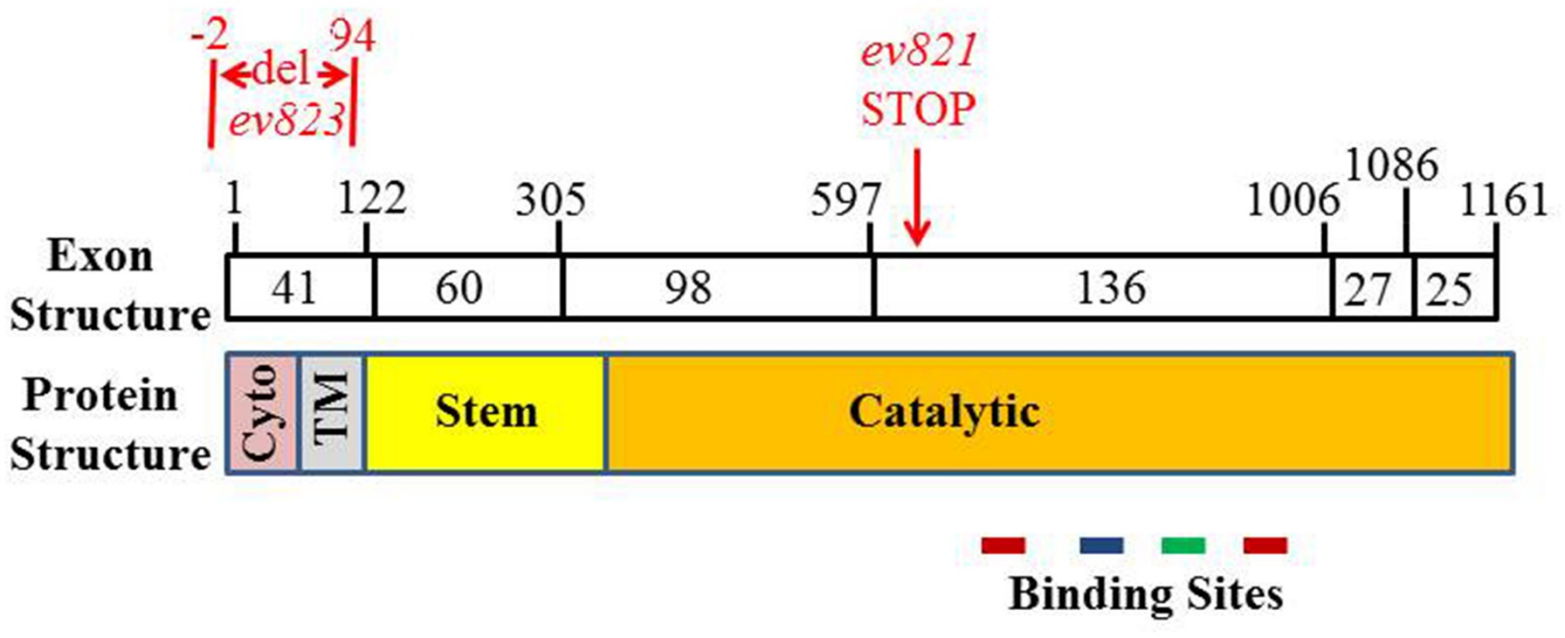
Organization of *ngat-1* coding sequence and encoded protein. Boxes represent cDNA exons whose approximate codon size is indicated within. Exon boundaries and the positions of the *ev821* point mutation (red) and the *ev823* deletion (red) are indicated above the exon boxes. The corresponding protein domain structure [48] is indicated below the exons (Cyto = Cytoplasmic domain, TM = transmembrane domain). Color coding for binding sites is as in **Fig 4**.

W02B12.11 encodes a 387-residue protein (**Figs 4 and 5**). The DNA sequence data indicates that the *ev821* mutant strain carries a T for C substitution creating a chain terminating stop codon for codon 209 in exon IV of the W02B12.11 gene. To confirm the identification of *ev821*, we generated a second allele of the *W02B12*.*11* gene using CRISPR-Cas9 gene editing [41] to target a deletion in the first exon of *ngat-1*. Candidate insertion/deletion strains were identified and a 96 base pair deletion (*ev823*) was verified that removed the sequence encoding nucleotides -2 to +94 with the A of ATG as position +1 of exon 1 (**Fig 5**). Thus the *ev823* mutant is predicted to encode a protein missing the normal initiator codon and the N-terminal 31 residues of *W02B12*.*11*. The next in-frame ATG codon is codon 89 within the Stem domain. Mutant *ngat-1*(*ev823)* animals grown at 25°C and analyzed by D.I.C. microscopy manifested posterior DTC phase 2 migration defects similar to those observed in *ngat-1(ev821)*, but at lower penetrance of 33% (n = 268) of animals, compared with 68% (n = 123) for *ev821* grown at 25°C. As with *ev821*, this phenotype was temperature sensitive, with the penetrance of VCPs reduced to 16% (n = 168) following growth at 20°C. These results strongly support the identification of *W02B12*.*11* as causal for the *ev821* and *ev823* phase 2 DTC migration defects. The finding that both an apparently strong, possibly null allele like *ev821*, and a weaker, possibly hypomorphic allele like *ev823* cause the same spectrum of phase 2 defects and are both ts for these phenotypes suggests that any reduction of *ngat-1/W02B12*.*11* gene activity reveals an underlying ts process or structure required for normal phase 2 DTC navigation.

The *W02B12*.*11* gene encodes a protein with sequence homology to the ß1-4- galactosyltransferase gene family (**Fig 4**). In these Golgi localized enzymes, a key residue in the catalytic domain determines if the enzyme utilizes UDP-galactose or UDP-N-acetylgalactosamine for the transfer reaction—with tyrosine and phenylalanine specifying galactose, and leucine or isoleucine specifying N-acetylgalactosaminyl transfer [45]. This key residue in *W02B12*.*11* is isoleucine (**Fig 4**)—characteristic of enzymes that transfer N-acetylgalactosamine; thus, we have renamed the gene *ngat-1* for ß1,4-N-acetylgalactosaminyltransferase.

The *ev821* chain-termination nonsense mutation at codon 209 predicts deletion of the C-terminal half of the catalytic domain (**Fig 5**), including key residues for metal ion binding, sugar donor and sugar acceptor interactions [45]. This mutation is therefore likely to be a null mutation. Only one other *C*. *elegans* gene, *bre-4*, encodes an enzyme with the same predicted activity as NGAT-1, but it functions in the intestine [46] and does not cause posterior DTC migration defects, nor does it enhance the DTC defects of *ngat-1(ev821)* (**S1 Table**).

### Loss of complex-type N-glycans causes DTC migration defects

**Fig 6:**
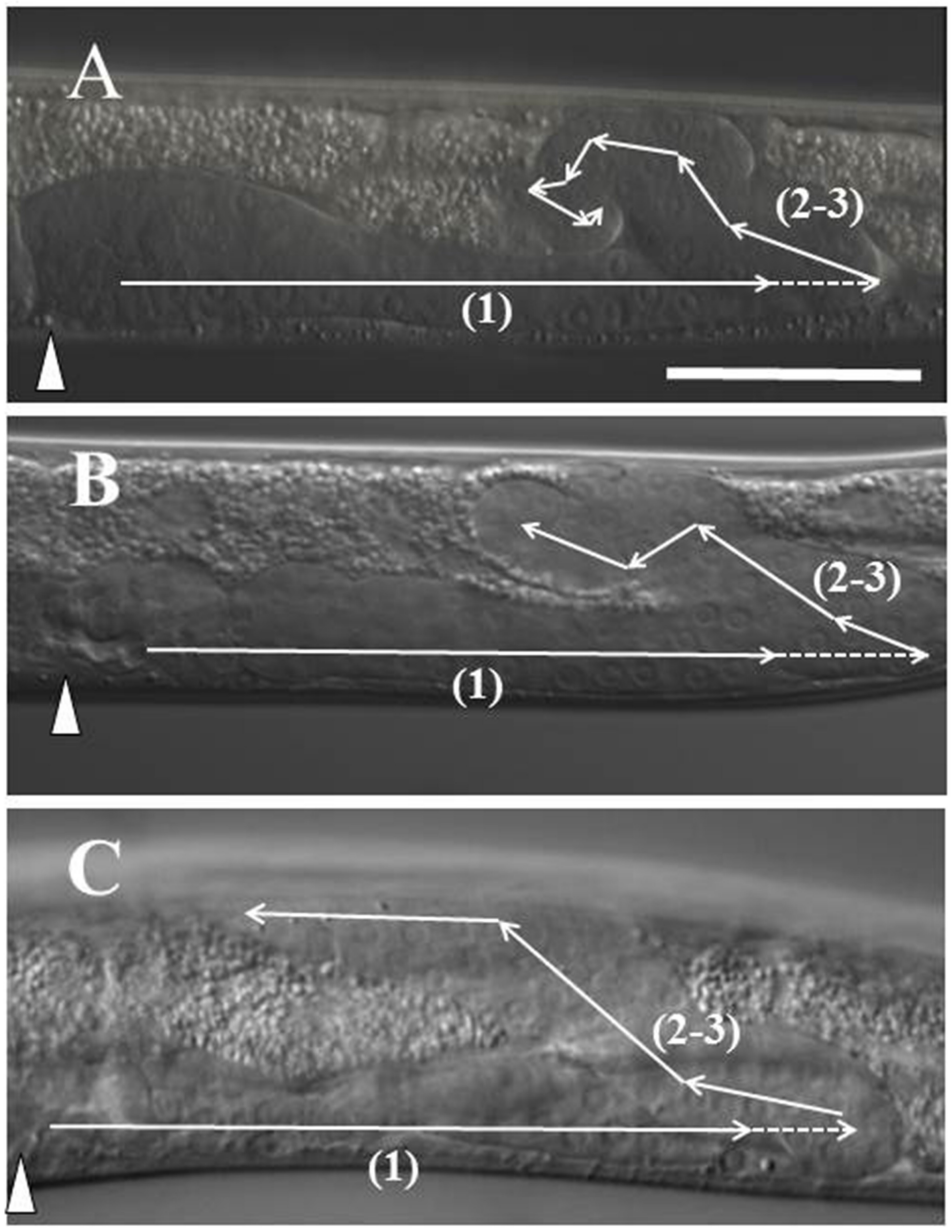
Common posterior gonad arm phenotypes of the *GnT1/Mgat1* triple null mutant. (**A-C**) Diagram ***iv*** errors of **Fig 2**are of various trajectories in *ngat-1* and the *GnT1* triple mutants following normal phase 1 navigation, three of which are shown here by D.I.C. microscopy. In these, following a normal phase 1, the posterior DTC changes direction often and seemingly randomly, but rarely away from mid-body, although the distal arm in these cases does not return to the mid-body region. Panel **C** shows a posterior gonad arm like that of diagram ***ii***. The return toward mid-body on the ventral side is usually shorter in the *GnT1* triple compared to the *ngat-1(ev821)* mutant. Premature phase 1 ventral-to-dorsal migrations were observed for 7 anterior DTCs of 130 *GnT1* triple mutant animals examined. Scale bar in panel **A** (for **A-C**) is 50 μm.

The evidence that *ngat-1* is required for normal DTC migration indicates that carbohydrate modifications play an important role in guided DTC migration. A possible role for the NGAT-1 enzyme may involve addition of N-acetylgalactosamine to complex N-glycans on one or more migration-relevant glycoproteins required for phase 2 DTC migration. If so, mutations that disrupt early steps in the elaboration of core glycans could block the synthesis of NGAT-1 dependent substrate structures and cause migration defects similar to those observed for *ev821*. *C*. *elegans* has three genes (*gly-12*,*-13*, and -*14*) encoding functional β-1,2-N-acetylglucosaminyltransferase enzymes (also known as GnTI or Mgat1) that catalyze the first step of N-glycan branching in the Golgi. Deletion of GnT1 activity in mice causes mid-gestation embryonic lethality [49, 50]. It was reported previously that a *gly-14(id48)III; gly-12(id47) gly-13(ok712)X* triple null mutant (putative *GnT1/Mgat1* null) of *C*. *elegans* has no obvious developmental defects (Zhu et al. 2004); however, careful examination of the gonad arms of triple mutant hermaphrodites grown at 25°C revealed a normal phase 1 trajectory with phase 2 migration errors in 114 of 130 (88%) of posterior DTCs examined. Among 56 of these animals that were also scored for anterior DTC migration errors, there were only 4 anterior DTC migration defects, 2 of which were phase 2 specific and one each phase 1 or phase 3 specific. The posterior DTC phase 2 errors in the triple mutant often manifested somewhat differently from the phase 2 errors of the *ngat-1(ev821)* mutant. In the *ngat-1* mutant, most gonad arms project back toward mid-body usually along the proximal arm of the gonad on the ventral body wall, however, in the *GnT1* triple mutant, more often than in the *ngat-1(ev821)* mutant, the posterior DTC at the end of phase 1 projected a short distance toward mid-body, then moved away from the ventral side making several short migrations (rarely oriented away from mid-body) separated by abrupt changes in direction before stopping at some distance from the mid-body region (**Fig 6A and 6B**). Changes in direction were usually accompanied by a bulbous protrusion in the girth of the gonad arm and occasional changes in sidedness of the DTC migration during phase 3. The migration back toward mid-body along the proximal gonad arm on the ventral side did not occur as frequently or to the same extent in the *GnT1* triple null mutant as in the *ngat-1* mutants (**Fig 6C**), thereby causing a lower penetrance (approximately 30%) of visible VCPs compared to the nearly 75% penetrance of VCPs in the *ngat-1* mutant animals. Furthermore, the phase 2 posterior DTC migration errors in *GnT1* triple null mutant animals grown at 25°C were more frequent (48 of 56) than those grown at 20°C (30 of 563), which were more frequent than those grown at 16°C (8 of 62) (Panel B in **S1 Fig**). Similar phase 2 migration errors, the same temperature-sensitivity, and similar differences in penetrance of posterior vs anterior DTC migration defects raise the possibility that NGAT-1 and the GnT1 enzymes act sequentially on the same N-glycoprotein target(s) to prevent posterior DTC migration errors, with the *ngat-1* defect being perhaps a slightly milder version of the *GnT1* triple mutant defects, which are more penetrant.

### Temperature sensitive period of *GnT1* triple null mutant

**Fig 7:**
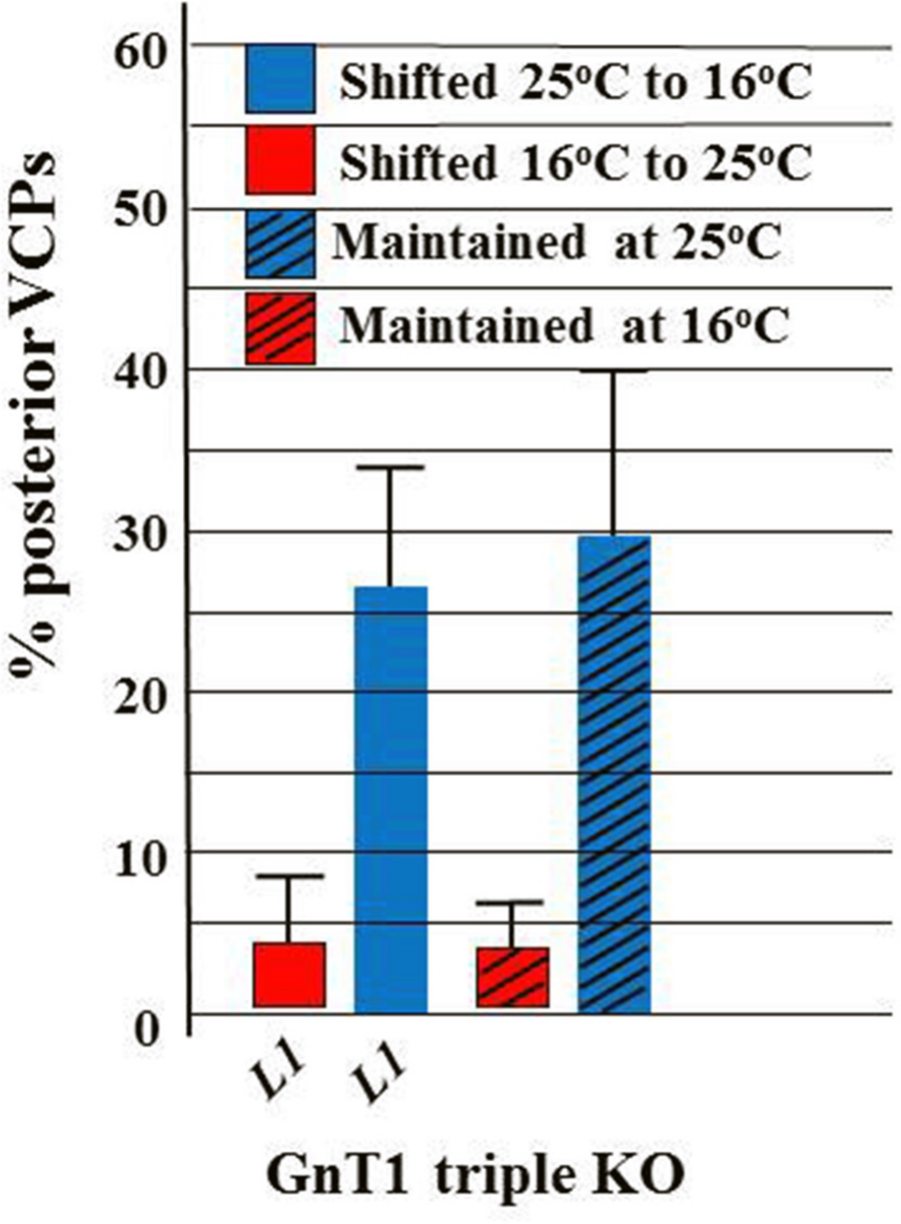
Temperature-sensitive period for *GnT1* triple mutant phase 2 DTC migration errors. A temperature shift experiment similar to that of **Fig 3**, but limited to shifting only L1s (based on the **Fig 3***ev821* results) was performed for the *GnT1* triple null mutant scored for all 3 kinds of VCPs by low magnification stereomicroscopy. Visible VCPs are not as frequent as phase 2 defects scored by D.I.C. in the *GnT1* triple mutant, probably because the distal arm return along the proximal arm is typically much shorter (creating a much smaller VCP) in the *GnT1* triple than in *ngat-1(ev821)*. Neither the *ngat-1(ev821)* nor the *GnT1* triple null mutant can prevent, reverse, or compensate a phase 2 DTC migration failure induced by embryonic growth at 25°C if shifted back to 16°C during the first larval (L1) phase, whereas embryonic growth at 16°C leads to largely normal DTC migration that cannot be reversed by a shift to 25°C during the first larval (L1) stage.

To further examine the idea that GnT1 and NGAT-1 may affect the same target(s) for glycosylation required for phase 2 DTC migration, we determined the approximate ts period of the *GnT1* triple knockout mutant by scoring posterior VCPs. From **Fig 7**it is clear that the *GnT1* triple null mutant strain cannot reverse a phase 2 DTC migration failure induced by embryonic growth at 25°C if shifted back to 16°C during the first larval L1 phase, whereas embryonic growth at 16°C leads to wild-type/normal DTC migration that can be only partially prevented by a shift to 25°C during the L1 stage. This shows that the ts period for phase 2 DTC migration revealed by the *GnT1* triple mutant probably ends during late embryogenesis or during the early L1 phase—just as the ts period revealed by the *ngat-1(ev821)* mutant does. This unusual phenotype suggests that both NGAT-1 and GnT1/Mgat1 acting in series may directly prevent, stabilize, reverse, or indirectly compensate for the same early developmental thermo-labile process (or structure) that leads to the much later 3^rd^ larval stage phase 2 navigation defects observed in both kinds of mutants, possibly by modifying glycan(s) on the same target glycoprotein(s) relevant to phase 2 DTC migration.

We decided to further examine the possibility that GnT1 and NGAT-1 act in the same pathway to prevent the temperature sensitivity of phase 2 DTC migration To do this, we generated the *ngat-1; gly-14; gly-12 gly-13* quadruple null mutant and compared its DTC migration defects to those of the *gly-14; gly-12 gly-13* triple null mutant. We expected an enhancement of the phase 2 DTC migration defects following growth at 20°C if the enzymes act in different pathways (e.g., via glycosylation of different targets) and no enhancement if they act in the same pathway (e.g., via glycosylation of the same target(s)). We found that the quadruple null mutant had nearly the same penetrance of posterior DTC phase 2 migration errors (42%, n = 65, measured by D.I.C.) as the triple null mutant (40%, n = 86) following growth in parallel at 20°C, while the *ngat-1(ev821)* segregant (see Materials and methods) grown in parallel manifested 46% (n = 63) phase 2 posterior DTC migration defects (**S2 Table)**. These results suggest that NGAT-1 and GnT1 enzyme activities function in the same pathway to prevent the early developmental temperature sensitivity of the posterior DTC phase 2 migration. Glycolipids in *C*. *elegans* can be modified by the NGAT-1 homolog BRE-4 [46], however, these quadruple mutant results effectively exclude glycolipids as relevant to the DTC migration phenotype observed in this study.

### *ngat-1*::*gfp* expression pattern

**Fig 8:**
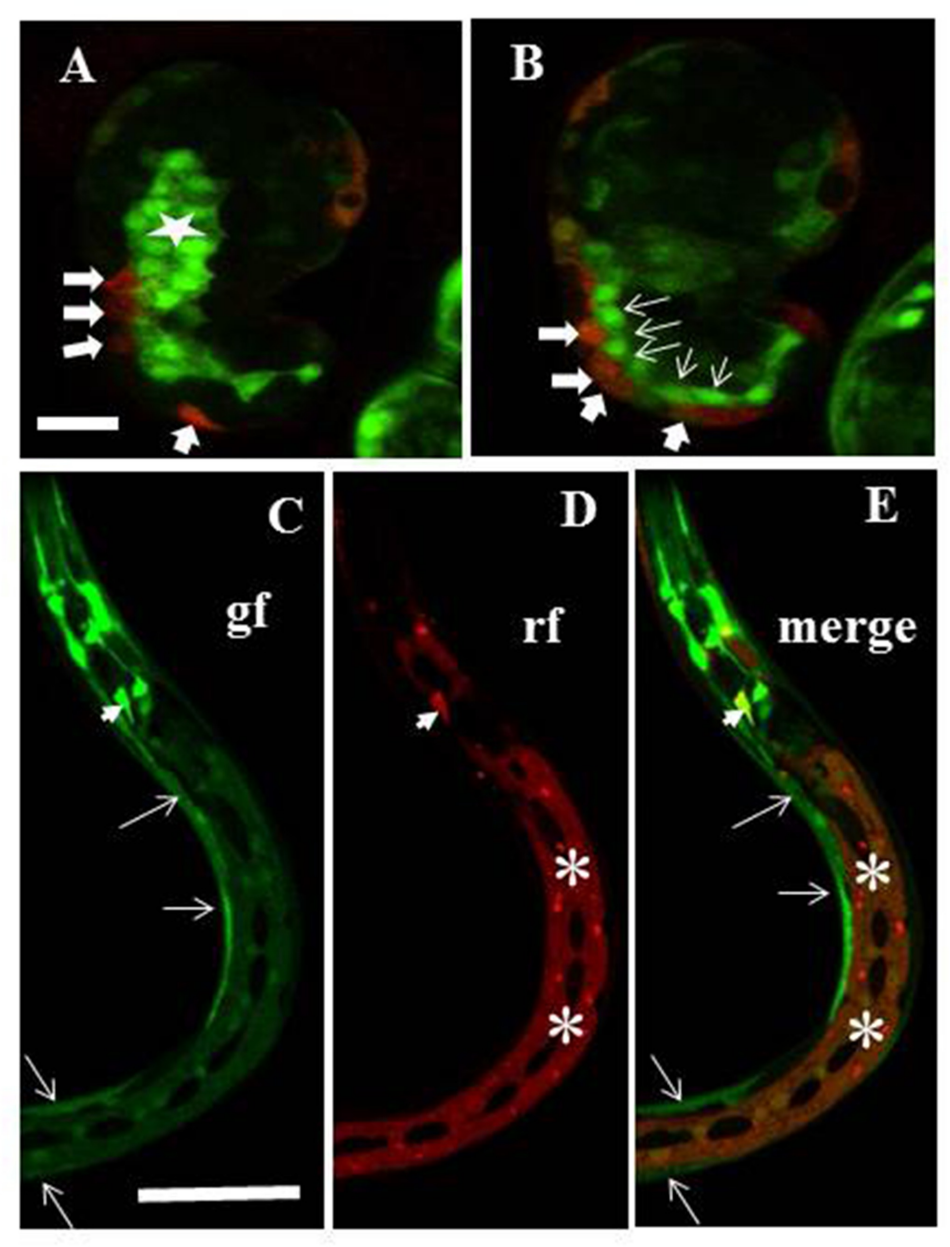
*ngat-1p*::*gfp* transcriptional reporter expression pattern. A chromosomally integrated *ngat-1p*::*gfp* transcriptional reporter strain was made as described in Materials and methods. A *dpy-7p*::*mCherry* transcriptional reporter was injected into the *ngat-1*::*gfp* integrated line to create a non-integrated marker for epidermal cells (see Materials and methods), which may not mark all epidermal cells in a given animal because of potential mitotic loss of the extrachromosomal array. (**A-B**) merge of *gfp* and *mCherry* signals showing presumptive *mCherry*-expressing epidermal cells (thick arrows) that co-express *gfp* (as indicated by orange tinge) plus presumptive muscle cells (thin arrows), and presumptive intestinal cells (star) that express only *ngat-1p*::*gfp* in a comma stage embryo. (**C-E**) The *ngat-1p*::*gfp* reporter is expressed in body wall muscles (arrows in **C** and **E**) and in lateral epidermis (asterisks in **D** and **E**) in all post-embryonic larval stages. Some unidentified elongated cells in the head also express *gfp* and at least one (arrowhead) also expresses *mCherry*. (Dark oval-shaped areas in the lateral epidermis are lateral seam cells). rf = red fluorescence channel; gf = green fluorescence channel. Scale bar in panel **A** (for **A** and **B**) is 12.5 μm. Scale bar in panel **C** (for **C-E**) is 100 μm.

An *ngat-1p*::*gfp* transcriptional reporter was made comprising 1592 bps of the putative 5’ upstream promoter region of *ngat-1* driving the *gfp* gene and a 3’ UTR of *unc-54*. Embryonic expression begins in the last third of embryonic development somewhat prior to the comma stage of embryogenesis (**Fig 8**). Embryonic expression was observed in many cells, many of which also express a *dpy-7* promoter-driven epidermal *mCherry* reporter, in presumptive BWMs, in intestinal precursors, in several unidentified accessory cells in the head, and in several additional unidentified embryonic cell types. GFP expression was also observed in the lateral epidermis, intestine, and body wall muscles of *ngat-1*::*gfp* larvae and adults (**Fig 8**). There was no obvious embryonic or post-embryonic expression in the gonad or DTCs, however, germline-specific expression is highly dependent on specific 3’ UTR binding proteins [51], so our use of the *unc-54* 3’ UTR in the above expression construct may prevent germline expression

Transcriptional reporters for the GnT1 encoding genes *gly-12*, *gly-13* and *gly-14* were reported by Chen et al. [32]. The *gly-12* reporter was expressed in all stages of development in many tissues including intestine, muscle, epidermis, other epithelial cells, and in ganglia in the head and tail region. The *gly-13* reporter was expressed from late embryogenesis to adulthood and was confined to intestinal cells in L1 larvae, but later roughly mimicked *gly-12* expression. The *gly-14* reporter was observed only in the intestine in animals ranging from L1 to adulthood. Expression was not detected in embryos, although significant levels of *gly-14* embryonic mRNA were detected.

### Cell/tissue-specific rescue

For cell type specific rescue experiments, we made animals transgenic for constructs in which cell type-specific heterologous promoters drive expression of *ngat-1(+)*—encoded by genomic DNA that includes all 6 exons and 698 bps of sequence downstream of the translational terminator codon. The *ev821* mutant DTC defects following growth at 25°C were largely rescued when *ngat-1*(+) expression was driven by the putatively muscle-specific *myo-3* promoter or by the putatively DTC-specific *lag-2* promoter (**S3 Fig, S3 Table**). This suggests that body wall muscle expression and DTC expression of *ngat-1(+)* are each sufficient to rescue the mutant DTC migration defects (however, see caveats in Discussion).

*ngat-1(ev821)* mutant animals transgenic for a construct of *ngat-1(+)* regulated by the intestine-specific *elt-2* promoter, were not significantly rescued their phase 2 DTC migration defects (**S3 Fig, S3 Table**). To examine the ability of hypodermal/epidermal expression to rescue, we used the *dpy-7* epidermis-specific promoter to drive *ngat-1(+)* expression, but were unable to recover viable transgenic progeny in several attempts at making them.

### NGAT-1 functions in parallel with the UNC-6/netrin pathway

To determine if NGAT-1 might function exclusively in the UNC-6/netrin signaling pathway required to prevent phase 2 DTC migration failures, we made an *ngat-1(ev821)*; *unc-6(ev400)* double mutant and quantified its VCP phenotype following growth at 25°C to compare with *unc-6(ev400)* and *ngat-1(ev821)* single mutant animals grown in parallel **(S4 Table)**. In this experiment the *ev821* homozygotes exhibited 39% netrin mutant-like phase 2 DTC migration failures identified by a full ventral clear patch extending from the vulva toward the tail (n = 105). There was an additional 35% penetrance of a partial clear patch posterior to and separate from the clear patch of the vulva. The *unc-6(ev400)* mutant grown in parallel under identical conditions exhibited a penetrance of 78% for these two kinds of VCPs (65% full and 13% partial VCPs, n = 101). *The unc-6(ev400); ngat-1(ev821)* double mutant grown under identical conditions exhibited a penetrance of 95% VCPs (84% full and 11% partial, n = 110), which is just statistically significant (p = 0.0423 by one-tail *z*-test for difference between 2 proportions) and almost exactly what is expected [78% + (74% x 22%) = 94%] if NGAT-1 has functions in parallel to the UNC-6/netrin signaling pathway that mediates phase 2 DTC migration. There was no apparent enhancement of the anterior phase 2 DTC VCPs of the *unc-6(ev400)* mutant by *ngat-1(ev821)* (**S4 Table**)—a result that is consistent with the finding that *ngat-1(ev821)* has little apparent effect on anterior DTC migration.

## Discussion

Phase 2 ventral-to-dorsal DTC migration across the lateral epidermis occurs during the late 3rd larval stage of development and this migration frequently fails in *unc-6*/netrin mutants and mutants of the netrin receptor encoding genes *unc-40* and *unc-5*. Our identification of weak alleles of *unc-40* in a screen for suppressors of axon guidance defects induced by ectopic expression of UNC-5 [52], supplied a sensitized genetic background in which to generate enhancer mutations that identify other genes required for DTC migration. The *ev821* mutation was identified as such an enhancer, but when isolated away from the weak *unc-40(ev543)* allele was found to induce DTC migration defects on its own. We cloned the *ev821* mutant gene by one step SNP/WGS and the *ev821* mutation was found to create a premature stop codon in the ORF for a putative N-acetyl-galactosaminyltransferase encoding gene we named *ngat-1*. Many, but not all of the *ngat-1(ev821)* mutant DTC migration defects were phase 2 DTC migration failures, nearly half of which were similar to those previously observed in mutants of *unc-5*, *unc-40* and *unc-6*, while other phase 2 migration errors varied in migration pattern, including those that began migrating toward mid-body on the ventral BWMs then migrated to the dorsal BWMs before reaching mid-body. Other relatively infrequent defects in *ev821* mutant DTC migration included DTCs meandering along the D/V axis on the ventral side as they migrate back toward the mid-body during the equivalent of phases 2 and 3 of migration. The DTC migration defects in *ngat-1(ev821)* (and *GnT1*/Mgat1 triple) mutants differ in several additional ways from the netrin mutant DTC migration defects. First, they appear to affect primarily posterior DTC migration and have few effects on anterior migration. This is somewhat unexpected, except that for unknown reasons, even in *unc-5*, *unc-6* and *unc-40* mutants the phase 2 navigational errors of the posterior DTC are more penetrant than the anterior DTC. However, anterior DTC migrations in the *ngat-1* and *GnT1* mutants might simply fall below some threshold for ventral to dorsal migration activity that is not present in the posterior DTC and not encountered in the netrin mutants. This seems unlikely, however, because one would have expected that any effect of *ngat-1* mutation on the phase 2 anterior DTC migration would have manifested in a genetic background sensitized by the absence of *unc-6* function, yet *ngat-1(ev821)* failed to enhance the anterior DTC defects (VCPs) of the *unc-6(ev400)* mutant. This suggests that *ngat-1*, unlike *unc-6*, has little if any role in anterior DTC migration (or is redundant with another unknown gene) and its function largely in posterior DTC migration may begin to account for known differences in the sensitivity of the two DTCs to mutations in other migration genes.

A second difference between the netrin mutants and *ngat-1(ev821)* is that the *ngat-1* mutant DTC migration defects are maternally rescued, that is, the homozygous mutant self-fertilization progeny of a heterozygous hermaphrodite parent rarely manifest phase 2 DTC navigation errors, whereas the self-fertilization progeny of these homozygotes do manifest a significant penetrance of these errors. Maternal rescue is known to occur for the majority of mutations in genes required for embryonic viability, which suggests that products of most maternally rescued genes are present when their function is needed during embryogenesis. Unlike most essential embryonic genes showing maternal rescue, the *ngat-1(+)* gene is required for a much later (post-embryonic) process leading to phase 2 DTC migration. A third difference between *ev821* and netrin mutants—the severe temperature sensitivity of the former and not the latter (**S5 Table**)–allowed us to estimate the developmental period in which subsequent phase 2 DTC migration is sensitive to temperature in the absence of NGAT-1 or, as we later showed, GnT1 function. Surprisingly, the ts period for *ngat-1* and *GnT1* mutants ends by the beginning of post-embryonic development or slightly thereafter in the first post-embryonic larval stage to allow a migration that occurs nearly 3 full larval stages later.

How can one reconcile the apparent early ts period with the late effect on DTC migration of missing NGAT-1 and GnT1 function? In principle, *ngat-1(ev821)* (and possibly one or more of the *gly* gene mutants) is temperature sensitive for synthesis of the corresponding gene product (which normally occurs during the ts period) or creates a ts NGAT-1 protein. However, the possibility of either of these scenarios for NGAT-1 or GnT1 is remote, given the high likelihood that both *ngat-1(ev821)* and the triple *gly-14; gly-12 gly-13* mutants are null for their respective NGAT-1 and GnT1 activities. This consideration suggests that NGAT-1 and GnT1 activities somehow prevent the innate temperature sensitivity of the phase 2 DTC migration, which has a ts period that ends by the L1 stage. NGAT-1 and GnT1 could do this by acting directly in a ts process or structure to prevent or reverse its thermolability (in which case the ts period could represent the period of NGAT-1 and GnT1 activity) or it could act in a separate process that bypasses or compensates for the ts deficiency in some other unknown way (in which case the ts period may not represent the time of NGAT-1 and GnT1 action). However, the great majority of maternally rescued genes with few exceptions [53] function during embryogenesis or perinatally, therefore it seems likely that NGAT-1 and GnT1 function during the innate ts period rather than closer to the time of the phase 2 migration they regulate. Distinguishing between these possibilities will require—as a first step—the identification and characterization of the target(s) of NGAT-1 and GnT1activity relevant to phase 2 DTC migration.

NGAT-1 and GnT1 are predicted by their enzymatic activity to modify one or more secreted glycoproteins. The ability of a *lag-2* promoter-driven *ngat-1(+)* construct to rescue mutant DTC migration defects suggests that a DTC-expressed glycoprotein can be modified by GnT1 and NGAT-1 to rescue the innate temperature sensitivity of the phase 2 DTC migration. The ability of body wall muscle (BWM) expressed NGAT-1 to rescue the *ngat-1(ev821)* DTC migration defects—even though BWM is not the substratum for the phase 2 migration–also suggests that a glycoconjugate target (possibly the same as the DTC-expressed target) of NGAT-1 activity can be secreted by BWMs to promote phase 2 migration at restrictive temperature, probably by incorporating into the gonad/DTC basement membrane. Precedent for this kind of autonomous and non-autonomous activity that promotes phase 2 DTC migration is found in MIG-17, which is made by body wall muscles and incorporates into the gonad basement membrane where it functions, but can also rescue a *mig-17* mutant DTC defects when expressed in the DTCs [12, 14]. An important caveat to the above interpretations is that *lag-*2 and *myo-3* reporters are not entirely specific to DTCs and muscle, respectively. For example, *myo-3* is also expressed by the gonad sheath cells [54], which help create the gonad/DTC basement membrane, and may conceivably also be expressed below limits of detection in the DTCs and *lag-2* is expressed in the ventral nerve cord and vulval precursor cells in the L3 stage [55]. Nonetheless, it is possible (as occurs for *mig-17*) that non-autonomous rescue by BWM and perhaps other tissue-specific expression of NGAT-1 does occur and possible that cell autonomous rescue can also occur.

One of our immediate goals is to identify potential targets for LacdiNAc modification by NGAT-1 in *C*. *elegans* and by related galactosyl transferases in mammals to begin to illuminate the *ngat-1* requirement for cell migration. When we started, we considered possible targets for NGAT-1 mediated glycosylation included N- or O- glycoproteins or glycolipids. The finding that *gly-14;gly-12 gly-13* triple mutant animals have DTC migration defects similar to those of *ngat-1* mutants (temperature-sensitive phase 2 errors largely limited to posterior DTCs with a similar early ts period) suggests that the migration-relevant NGAT-1 target(s) is the same N-glycoprotein(s) as the GnT1 target(s). The nearly identical penetrance of phase 2 DTC migration defects in the *gly-14; gly-12 gly-13* triple null and the *ngat-1; gly-14*, *gly-12 gly-13* quadruple null mutant suggests that the GnT1 activity encoded by the 3 *gly* genes and the activity encoded by *ngat-1* function in the same glycosylation pathway, which strongly supports the idea that GnT1 activity creates a N-glycan modification that serves as a substrate for NGAT-1-mediated LacdiNAc modification. Mass spectrometry analysis may be required to confirm this possibility, which if true would suggest that it is not the absence of GnT1 derived core glycan structures *per se* that cause the phase 2 DTC migration defects, but more likely the absence of the terminal LacdiNAc modification dependent upon these core glycan structures that is largely, but not totally, responsible for these defects.

### The *ngat-1* null mutant DTC migration phenotype differs from other glycosylation- associated gene mutants

Other glycogene mutants affecting DTC migration like *cogc-1*, *cogc-3*, *mig-22* and *mig-23* are likely to cause severe but unpredictable alterations of glycan chains of many proteins. These all appear to cause a meandering back to mid-body DTC migration pattern following an apparently normal phase 1 migration. The *ngat-1* mutations on the other hand are predicted to cause a very specific alteration in glycan structure (absence of a glycan-terminal LacdiNAc modification) which in this case leads to a fairly specific defect in DTC migration–phase 2-specific migration errors, which are frequent in the *ngat-1* mutants but not an obvious frequent phenotype observed in other glycogene mutants. This phenotype in *ngat-1* mutants does resemble the DTC phase 2 errors that are characteristic of netrin signaling mutants, however, there are some variants, especially DTCs that following phase 1 appear to return partially toward mid-body along the ventral BWMs before turning dorsally. The double mutant analysis indicates that NGAT-1 (and by inference GnT1) has navigation functions independent of the netrin signaling pathway as might be expected of a gene affecting the substratum for phase 2 DTC migration (the lateral epidermis for phase 2 migration), which should be necessary for both netrin dependent and netrin independent migration.

### Potential vertebrate roles

The *ngat-1* gene and its targets represent a potential causal link between a specific glyco-epitope (LacdiNAc) and cell migration. The discovery of glycogene mutants with cell migration defects predicted to cause deficits in LacdiNAc modification of glycoproteins has an interesting parallel to reports that the LacdiNAc epitope in mammals plays a role in cancer progression and metastasis [56–60]. Furthermore, one of us (JWD) has reported previously that deficiencies in one or more vertebrate enzymes of the N-glycan branching pathway (comprising Mgat1/GnT1, alpha-mannosidase II/ManII, Mgat2, Mgat4, and Mgat5 activity—see [61]) modifies tumor growth and reduces cell migration, invasion and metastasis [29, 61–63].

The predicted absence of a specific glyco-epitope (LacdiNAc) in *ngat-1* and *Mgat1/GnT1* (*gly12*, *-13*, *-14*) mutants that causes a specific cell migration defect and the existence of reagents that detect this epitope [64, 65] suggest it should be possible to biochemically identify the target(s) of NGAT-1 and GnT1 mediated glycosylation required for normal DTC phase 2 pathfinding, which are presumably present in both BWMs and DTCs as determined by cell type specific rescue experiments of the *ngat-1* mutant. The link between LacdiNAc modification and tumor progression further suggests that a genetic dissection of this mechanism in *C*. *elegans* and analysis of mammalian homologs of the relevant target genes identified in this genetic model could have translational potential. The maternal effect and later manifestation of a cell migration phenotype also suggests the possibility that NGAT-1 related enzymes in mammals might mediate a previously unrecognized perinatal function that contributes to later onset human health issues.

## Supporting information

S1 Fig Temperature-sensitivity of mutant DTC phase 2 migration defects. Bars indicate the penetrance of DTC phase 2 errors when grown >2 generations at the indicated temperature. (**A**) *ngat-1(ev821)* posterior DTC migration defects were scored as VCPs by stereomicroscopy, whereas, (**B**) *GnT1*/*gly-14; gly-12 gly-13* mutant DTC defects were scored by D.I.C. microscopy. Error bars represent z-test based 95% confidence intervals for a proportion.
(TIF)

S2 Fig Breakdown of DTC migration navigation errors of Fig 3 results. (**A**) Bars shows the penetrance of phase 2 posterior DTC migration failures of the types (DTC-1 to DTC-6) represented diagrammatically in panel **B**. L1-L4 = larval stages 1–4. Note that in a different plot of these data, shown in **Fig 3**, the DTC-5 category is not included as phase 2 errors because many similar trajectories were later found in wild-type animals grown at 25°C.
(TIF)

S3 Fig Gene and cell/tissue-specific rescue of the *ngat-1(ev821)* DTC migration defects. Constructs carrying *ngat-1(+)* driven by different regulatory regions were made as described in Materials and methods. These include **1)** *ngat-1(+)* in fosmid WRM0637aH09, **2)** a 4.3 PCR product encompassing *W02B12*.*11*, **3)** *ngat-1(+)* driven by body wall muscle specific promoter *myo-3p*, 4) DTC-specific promoter *lag-2*, and intestine-specific promoter *elt-2* (see Materials and methods). These results show that *W02B12*.*11/ngat-1(+)* expression in body wall muscles or in DTCs (see caveat in Discussion) is largely sufficient to rescue the *ngat-1(ev821)* mutant DTC migration defects of animals grown at 25°C, whereas, expression in embryonic intestine by the *elt-2* promoter does not rescue these defects. Error bars represent z-test based 95% confidence intervals for a proportion.
(TIF)

S1 Table *ngat-1(ev821)* is not enhanced by a *bre-4* putative null mutation for DTC migration defects. ^1^An *ngat-1(ev821); bre-4(ok3167)* double mutant—constructed by standard methods—was grown in parallel with both single mutants for two generations at 23°C before monitoring VCPs in L4 animals. Shown are number (#) of gonad arms scored and percentage (%) of animals with given VCP phenotype.
(TIF)

S2 Table *ngat-1* putative null mutation and a *GnT1* null mutation do not enhance each other for DTC navigation errors. ^1^Number (#) and percentage (%) of animals with phase 2 posterior DTC migration defects.
^2^Two of the 3 anterior DTC navigation errors (in the triple and quadruple mutants) were premature phase 1 migration toward the dorsal side and 1 was a phase 2 error.
^3^One anterior DTC migrated along a normal 3 phase trajectory except on the left instead of the right side epidermis in the triple mutant. There was 1, 1 and 3 otherwise normally guided but wrong-sided posterior DTC migrations in the triple, quadruple, and single mutant, respectively.
(TIF)

S3 Table DNAs used for transgene constructs and injections. (TIF)

S4 Table An *unc-6* null allele is enhanced by *ngat-1(ev821)* for DTC VCPs. ^1^An *ngat-1(ev821); unc-6(ev400)* double mutant—constructed by standard methods—was grown in parallel with both single mutants for two generations at 25°C before scoring VCPs in L4 animals. Ant. = Anterior DTC; Post. = Posterior DTC; Full = full and near-full, Part. = Partial; VCP = Ventral Clear Patch, # = number of gonad arms scored. % = percentage of animals with a given VCP phenotype.
(TIF)

S5 Table *unc-6(ev400)* animals are not temperature-sensitive for DTC migration defects. ^1^Number (#) of gonad arms scored following growth for two generations at 16°C or 25°C and percentage (%) of animals with a given VCP phenotype.
^2^ p = 0.66 by 2-tailed z-test.
^3^ p = 0.47 by 2-tailed z-test.
(TIF)

## References

[1] Hedgecock EM, Culotti JG, Hall DH. The unc-5, unc-6, and unc-40 genes guide circumferential migrations of pioneer axons and mesodermal cells on the epidermis in C. elegans. Neuron. 1990;4(1):61–85. Epub 1990/01/01. . 2310575 10.1016/0896-6273(90)90444-k

[2] Merz DC, Zheng H, Killeen MT, Krizus A, Culotti JG. Multiple signaling mechanisms of the UNC-6/netrin receptors UNC-5 and UNC-40/DCC in vivo. Genetics. 2001;158(3):1071–80. Epub 2001/07/17. ; PubMed Central PMCID: PMC1461735. 11454756 10.1093/genetics/158.3.1071 PMC1461735

[3] Su M, Merz DC, Killeen MT, Zhou Y, Zheng H, Kramer JM, et al Regulation of the UNC-5 netrin receptor initiates the first reorientation of migrating distal tip cells in Caenorhabditis elegans. Development. 2000;127(3):585–94. Epub 2000/01/13. . 10631179 10.1242/dev.127.3.585

[4] Saied-Santiago K, Townley RA, Attonito JD, da Cunha DS, Diaz-Balzac CA, Tecle E, et al Coordination of Heparan Sulfate Proteoglycans with Wnt Signaling To Control Cellular Migrations and Positioning in Caenorhabditis elegans. Genetics. 2017 Epub 2017/06/04. doi: 10.1534/genetics.116.198739 . 28576860 10.1534/genetics.116.198739 PMC5560800

[5] Diaz-Balzac CA, Lazaro-Pena MI, Tecle E, Gomez N, Bulow HE. Complex cooperative functions of heparan sulfate proteoglycans shape nervous system development in Caenorhabditis elegans. G3 (Bethesda). 2014;4(10):1859–70. Epub 2014/08/08. doi: 10.1534/g3.114.012591 ; PubMed Central PMCID: PMC4199693. 25098771 10.1534/g3.114.012591 PMC4199693

[6] Merz DC, Alves G, Kawano T, Zheng H, Culotti JG. UNC-52/perlecan affects gonadal leader cell migrations in C. elegans hermaphrodites through alterations in growth factor signaling. Dev Biol. 2003;256(1):173–86. Epub 2003/03/26. . 12654300 10.1016/s0012-1606(03)00014-9

[7] Schwabiuk M, Coudiere L, Merz DC. SDN-1/syndecan regulates growth factor signaling in distal tip cell migrations in C. elegans. Dev Biol. 2009;334(1):235–42. Epub 2009/07/28. doi: 10.1016/j.ydbio.2009.07.020 . 19631636 10.1016/j.ydbio.2009.07.020

[8] Suzuki N, Toyoda H, Sano M, Nishiwaki K. Chondroitin acts in the guidance of gonadal distal tip cells in C. elegans. Dev Biol. 2006;300(2):635–46. Epub 2006/09/20. doi: 10.1016/j.ydbio.2006.08.037 . 16982046 10.1016/j.ydbio.2006.08.037

[9] Kinnunen T, Huang Z, Townsend J, Gatdula MM, Brown JR, Esko JD, et al Heparan 2-O-sulfotransferase, hst-2, is essential for normal cell migration in Caenorhabditis elegans. Proc Natl Acad Sci U S A. 2005;102(5):1507–12. Epub 2005/01/27. doi: 10.1073/pnas.0401591102 ; PubMed Central PMCID: PMC547812. 15671174 10.1073/pnas.0401591102 PMC547812

[10] Xu X, Rongali SC, Miles JP, Lee KD, Lee M. pat-4/ILK and unc-112/Mig-2 are required for gonad function in Caenorhabditis elegans. Exp Cell Res. 2006;312(9):1475–83. Epub 2006/02/16. doi: 10.1016/j.yexcr.2006.01.006 . 16476426 10.1016/j.yexcr.2006.01.006

[11] Lee M, Cram EJ, Shen B, Schwarzbauer JE. Roles for beta(pat-3) integrins in development and function of Caenorhabditis elegans muscles and gonads. J Biol Chem. 2001;276(39):36404–10. Epub 2001/07/27. doi: 10.1074/jbc.M105795200 . 11473126 10.1074/jbc.M105795200

[12] Nishiwaki K, Hisamoto N, Matsumoto K. A metalloprotease disintegrin that controls cell migration in Caenorhabditis elegans. Science. 2000;288(5474):2205–8. Epub 2000/06/24. . 10864868 10.1126/science.288.5474.2205

[13] Blelloch R, Anna-Arriola SS, Gao D, Li Y, Hodgkin J, Kimble J. The gon-1 gene is required for gonadal morphogenesis in Caenorhabditis elegans. Dev Biol. 1999;216(1):382–93. Epub 1999/12/10. doi: 10.1006/dbio.1999.9491 . 10588887 10.1006/dbio.1999.9491

[14] Ihara S, Nishiwaki K. Prodomain-dependent tissue targeting of an ADAMTS protease controls cell migration in Caenorhabditis elegans. EMBO J. 2007;26(11):2607–20. Epub 2007/05/12. doi: 10.1038/sj.emboj.7601718 ; PubMed Central PMCID: PMC1888677. 17491590 10.1038/sj.emboj.7601718 PMC1888677

[15] Nishiwaki K, Kubota Y, Chigira Y, Roy SK, Suzuki M, Schvarzstein M, et al An NDPase links ADAM protease glycosylation with organ morphogenesis in C. elegans. Nat Cell Biol. 2004;6(1):31–7. Epub 2003/12/23. doi: 10.1038/ncb1079 . 14688791 10.1038/ncb1079

[16] Kawano T, Zheng H, Merz DC, Kohara Y, Tamai KK, Nishiwaki K, et al C. elegans mig-6 encodes papilin isoforms that affect distinct aspects of DTC migration, and interacts genetically with mig-17 and collagen IV. Development. 2009;136(9):1433–42. Epub 2009/03/20. doi: 10.1242/dev.028472 ; PubMed Central PMCID: PMC2674254. 19297413 10.1242/dev.028472 PMC2674254

[17] Kubota Y, Sano M, Goda S, Suzuki N, Nishiwaki K. The conserved oligomeric Golgi complex acts in organ morphogenesis via glycosylation of an ADAM protease in C. elegans. Development. 2006;133(2):263–73. Epub 2005/12/16. doi: 10.1242/dev.02195 . 16354716 10.1242/dev.02195

[18] Levy-Strumpf N, Culotti JG. Netrins and Wnts function redundantly to regulate antero-posterior and dorso-ventral guidance in C. elegans. PLoS Genet. 2014;10(6):e1004381 Epub 2014/06/06. doi: 10.1371/journal.pgen.1004381 ; PubMed Central PMCID: PMC4046927. 24901837 10.1371/journal.pgen.1004381 PMC4046927

[19] Itoh B, Hirose T, Takata N, Nishiwaki K, Koga M, Ohshima Y, et al SRC-1, a non-receptor type of protein tyrosine kinase, controls the direction of cell and growth cone migration in C. elegans. Development. 2005;132(23):5161–72. Epub 2005/10/28. doi: 10.1242/dev.02103 . 16251208 10.1242/dev.02103

[20] Lucanic M, Kiley M, Ashcroft N, L'Etoile N, Cheng HJ. The Caenorhabditis elegans P21-activated kinases are differentially required for UNC-6/netrin-mediated commissural motor axon guidance. Development. 2006;133(22):4549–59. Epub 2006/10/20. doi: 10.1242/dev.02648 . 17050621 10.1242/dev.02648

[21] Lundquist EA, Reddien PW, Hartwieg E, Horvitz HR, Bargmann CI. Three C. elegans Rac proteins and several alternative Rac regulators control axon guidance, cell migration and apoptotic cell phagocytosis. Development. 2001;128(22):4475–88. Epub 2001/11/21. . 11714673 10.1242/dev.128.22.4475

[22] Soto MC, Qadota H, Kasuya K, Inoue M, Tsuboi D, Mello CC, et al The GEX-2 and GEX-3 proteins are required for tissue morphogenesis and cell migrations in C. elegans. Genes Dev. 2002;16(5):620–32. Epub 2002/03/06. doi: 10.1101/gad.955702 ; PubMed Central PMCID: PMC155352. 11877381 10.1101/gad.955702 PMC155352

[23] Cabello J, Neukomm LJ, Gunesdogan U, Burkart K, Charette SJ, Lochnit G, et al The Wnt pathway controls cell death engulfment, spindle orientation, and migration through CED-10/Rac. PLoS Biol. 2010;8(2):e1000297 Epub 2010/02/04. doi: 10.1371/journal.pbio.1000297 ; PubMed Central PMCID: PMC2814829. 20126385 10.1371/journal.pbio.1000297 PMC2814829

[24] Cram EJ, Shang H, Schwarzbauer JE. A systematic RNA interference screen reveals a cell migration gene network in C. elegans. J Cell Sci. 2006;119(Pt 23):4811–8. Epub 2006/11/09. doi: 10.1242/jcs.03274 . 17090602 10.1242/jcs.03274

[25] Varki A, Kannagi R, Toole BP. Glycosylation Changes in Cancer. In: Varki A, Cummings RD, Esko JD, Freeze HH, Stanley P, Bertozzi CR, et al, editors. Essentials of Glycobiology. 2nd ed. Cold Spring Harbor (NY)2009. 20301279

[26] Deprez P, Gautschi M, Helenius A. More than one glycan is needed for ER glucosidase II to allow entry of glycoproteins into the calnexin/calreticulin cycle. Mol Cell. 2005;19(2):183–95. Epub 2005/07/26. doi: 10.1016/j.molcel.2005.05.029 . 16039588 10.1016/j.molcel.2005.05.029

[27] Warren CE, Krizus A, Dennis JW. Complementary expression patterns of six nonessential Caenorhabditis elegans core 2/I N-acetylglucosaminyltransferase homologues. Glycobiology. 2001;11(11):979–88. Epub 2001/12/18. . 11744632 10.1093/glycob/11.11.979

[28] Warren CE, Krizus A, Roy PJ, Culotti JG, Dennis JW. The Caenorhabditis elegans gene, gly-2, can rescue the N-acetylglucosaminyltransferase V mutation of Lec4 cells. J Biol Chem. 2002;277(25):22829–38. Epub 2002/04/09. doi: 10.1074/jbc.M201390200 . 11937505 10.1074/jbc.M201390200

[29] Lau KS, Dennis JW. N-Glycans in cancer progression. Glycobiology. 2008;18(10):750–60. Epub 2008/08/15. doi: 10.1093/glycob/cwn071 . 18701722 10.1093/glycob/cwn071

[30] Contessa JN, Bhojani MS, Freeze HH, Ross BD, Rehemtulla A, Lawrence TS. Molecular imaging of N-linked glycosylation suggests glycan biosynthesis is a novel target for cancer therapy. Clin Cancer Res. 2010;16(12):3205–14. Epub 2010/04/24. doi: 10.1158/1078-0432.CCR-09-3331 ; PubMed Central PMCID: PMC3413408. 20413434 10.1158/1078-0432.CCR-09-3331 PMC3413408

[31] Ohtsubo K, Marth JD. Glycosylation in cellular mechanisms of health and disease. Cell. 2006;126(5):855–67. Epub 2006/09/09. doi: 10.1016/j.cell.2006.08.019 . 16959566 10.1016/j.cell.2006.08.019

[32] Chen S, Zhou S, Sarkar M, Spence AM, Schachter H. Expression of three Caenorhabditis elegans N-acetylglucosaminyltransferase I genes during development. J Biol Chem. 1999;274(1):288–97. Epub 1998/12/29. . 9867843 10.1074/jbc.274.1.288

[33] Brenner S. The genetics of Caenorhabditis elegans. Genetics. 1974;77(1):71–94. Epub 1974/05/01. ; PubMed Central PMCID: PMC1213120. 4366476 10.1093/genetics/77.1.71 PMC1213120

[34] Wood WB. The Nematode Caenorhabditis elegans. Cold Spring Harbour, NY: Cold Spring Harbor Laboratory Press; 1988.

[35] Zhu S, Hanneman A, Reinhold VN, Spence AM, Schachter H. Caenorhabditis elegans triple null mutant lacking UDP-N-acetyl-D-glucosamine:alpha-3-D-mannoside beta1,2-N-acetylglucosaminyltransferase I. Biochem J. 2004;382(Pt 3):995–1001. Epub 2004/07/02. doi: 10.1042/BJ20040793 ; PubMed Central PMCID: PMC1133976. 15228383 10.1042/BJ20040793 PMC1133976

[36] Hodgkin J, Horvitz HR, Brenner S. Nondisjunction Mutants of the Nematode CAENORHABDITIS ELEGANS. Genetics. 1979;91(1):67–94. Epub 1979/01/01. ; PubMed Central PMCID: PMC1213932. 17248881 10.1093/genetics/91.1.67 PMC1213932

[37] Minevich G, Park DS, Blankenberg D, Poole RJ, Hobert O. CloudMap: a cloud-based pipeline for analysis of mutant genome sequences. Genetics. 2012;192(4):1249–69. Epub 2012/10/12. doi: 10.1534/genetics.112.144204 ; PubMed Central PMCID: PMC3512137. 23051646 10.1534/genetics.112.144204 PMC3512137

[38] Sambrook J, Fristsch E. F., and Maniatis T. Molecular Cloning: A Laboratory Manual: Cold Spring Harbor Laboratory Press; 1989.

[39] L'Etoile ND, Bargmann CI. Olfaction and odor discrimination are mediated by the C. elegans guanylyl cyclase ODR-1. Neuron. 2000;25(3):575–86. Epub 2000/04/25. . 10774726 10.1016/s0896-6273(00)81061-2

[40] Mello C, Fire A. DNA transformation. Methods Cell Biol. 1995;48:451–82. Epub 1995/01/01. . 8531738

[41] Dickinson DJ, Ward JD, Reiner DJ, Goldstein B. Engineering the Caenorhabditis elegans genome using Cas9-triggered homologous recombination. Nat Methods. 2013;10(10):1028–34. Epub 2013/09/03. doi: 10.1038/nmeth.2641 ; PubMed Central PMCID: PMC3905680. 23995389 10.1038/nmeth.2641 PMC3905680

[42] Brokate-Llanos AM, Monje JM, Murdoch Pdel S, Munoz MJ. Developmental defects in a Caenorhabditis elegans model for type III galactosemia. Genetics. 2014;198(4):1559–69. Epub 2014/10/10. doi: 10.1534/genetics.114.170084 ; PubMed Central PMCID: PMC4256771. 25298520 10.1534/genetics.114.170084 PMC4256771

[43] Burke SL, Hammell M, Ambros V. Robust Distal Tip Cell Pathfinding in the Face of Temperature Stress Is Ensured by Two Conserved microRNAS in Caenorhabditis elegans. Genetics. 2015;200(4):1201–18. Epub 2015/06/17. doi: 10.1534/genetics.115.179184 ; PubMed Central PMCID: PMC4574240. 26078280 10.1534/genetics.115.179184 PMC4574240

[44] Doitsidou M, Poole RJ, Sarin S, Bigelow H, Hobert O. C. elegans mutant identification with a one-step whole-genome-sequencing and SNP mapping strategy. PLoS One. 2010;5(11):e15435 Epub 2010/11/17. doi: 10.1371/journal.pone.0015435 ; PubMed Central PMCID: PMC2975709. 21079745 10.1371/journal.pone.0015435 PMC2975709

[45] Ramakrishnan B, Qasba PK. Role of a single amino acid in the evolution of glycans of invertebrates and vertebrates. J Mol Biol. 2007;365(3):570–6. Epub 2006/11/07. doi: 10.1016/j.jmb.2006.10.034 ; PubMed Central PMCID: PMC1850938. 17084860 10.1016/j.jmb.2006.10.034 PMC1850938

[46] Griffitts JS, Huffman DL, Whitacre JL, Barrows BD, Marroquin LD, Muller R, et al Resistance to a bacterial toxin is mediated by removal of a conserved glycosylation pathway required for toxin-host interactions. J Biol Chem. 2003;278(46):45594–602. Epub 2003/08/29. doi: 10.1074/jbc.M308142200 . 12944392 10.1074/jbc.M308142200

[47] McWilliam H, Li W, Uludag M, Squizzato S, Park YM, Buso N, et al Analysis Tool Web Services from the EMBL-EBI. Nucleic Acids Res. 2013;41(Web Server issue):W597–600. Epub 2013/05/15. doi: 10.1093/nar/gkt376 ; PubMed Central PMCID: PMC3692137. 23671338 10.1093/nar/gkt376 PMC3692137

[48] Qasba PK, Ramakrishnan B, Boeggeman E. Structure and function of beta -1,4-galactosyltransferase. Curr Drug Targets. 2008;9(4):292–309. Epub 2008/04/09. ; PubMed Central PMCID: PMC2365515. 18393823 10.2174/138945008783954943 PMC2365515

[49] Ioffe E, Stanley P. Mice lacking N-acetylglucosaminyltransferase I activity die at mid-gestation, revealing an essential role for complex or hybrid N-linked carbohydrates. Proc Natl Acad Sci U S A. 1994;91(2):728–32. Epub 1994/01/18. ; PubMed Central PMCID: PMC43022. 8290590 10.1073/pnas.91.2.728 PMC43022

[50] Metzler M, Gertz A, Sarkar M, Schachter H, Schrader JW, Marth JD. Complex asparagine-linked oligosaccharides are required for morphogenic events during post-implantation development. EMBO J. 1994;13(9):2056–65. Epub 1994/05/01. ; PubMed Central PMCID: PMC395055. 8187759 10.1002/j.1460-2075.1994.tb06480.x PMC395055

[51] Pushpa K, Kumar GA, Subramaniam K. Translational Control of Germ Cell Decisions. Results Probl Cell Differ. 2017;59:175–200. Epub 2017/03/02. doi: 10.1007/978-3-319-44820-6_6 . 28247049 10.1007/978-3-319-44820-6_6 PMC5985952

[52] Colavita A, Culotti JG. Suppressors of ectopic UNC-5 growth cone steering identify eight genes involved in axon guidance in Caenorhabditis elegans. Dev Biol. 1998;194(1):72–85. Epub 1998/03/14. doi: 10.1006/dbio.1997.8790 . 9473333 10.1006/dbio.1997.8790

[53] Hekimi S, Boutis P, Lakowski B. Viable maternal-effect mutations that affect the development of the nematode Caenorhabditis elegans. Genetics. 1995;141(4):1351–64. Epub 1995/12/01. ; PubMed Central PMCID: PMC1206872. 8601479 10.1093/genetics/141.4.1351 PMC1206872

[54] Ono K, Ono S. Two distinct myosin II populations coordinate ovulatory contraction of the myoepithelial sheath in the Caenorhabditis elegans somatic gonad. Mol Biol Cell. 2016;27(7):1131–42. Epub 2016/02/13. doi: 10.1091/mbc.E15-09-0648 ; PubMed Central PMCID: PMC4814220. 26864628 10.1091/mbc.E15-09-0648 PMC4814220

[55] Chen N, Greenwald I. The lateral signal for LIN-12/Notch in C. elegans vulval development comprises redundant secreted and transmembrane DSL proteins. Dev Cell. 2004;6(2):183–92. Epub 2004/02/13. . 14960273 10.1016/s1534-5807(04)00021-8

[56] Huang J, Liang JT, Huang HC, Shen TL, Chen HY, Lin NY, et al Beta1,4-N-acetylgalactosaminyltransferase III enhances malignant phenotypes of colon cancer cells. Mol Cancer Res. 2007;5(6):543–52. Epub 2007/06/21. doi: 10.1158/1541-7786.MCR-06-0431 . 17579116 10.1158/1541-7786.MCR-06-0431

[57] Che MI, Huang J, Hung JS, Lin YC, Huang MJ, Lai HS, et al beta1, 4-N-acetylgalactosaminyltransferase III modulates cancer stemness through EGFR signaling pathway in colon cancer cells. Oncotarget. 2014;5(11):3673–84. Epub 2014/07/09. doi: 10.18632/oncotarget.1981 ; PubMed Central PMCID: PMC4116512. 25003232 10.18632/oncotarget.1981 PMC4116512

[58] Hirano K, Matsuda A, Shirai T, Furukawa K. Expression of LacdiNAc groups on N-glycans among human tumors is complex. Biomed Res Int. 2014;2014:981627 Epub 2014/07/09. doi: 10.1155/2014/981627 ; PubMed Central PMCID: PMC4066867. 25003135 10.1155/2014/981627 PMC4066867

[59] Hsu WM, Che MI, Liao YF, Chang HH, Chen CH, Huang YM, et al B4GALNT3 expression predicts a favorable prognosis and suppresses cell migration and invasion via beta(1) integrin signaling in neuroblastoma. Am J Pathol. 2011;179(3):1394–404. Epub 2011/07/12. doi: 10.1016/j.ajpath.2011.05.025 ; PubMed Central PMCID: PMC3157223. 21741930 10.1016/j.ajpath.2011.05.025 PMC3157223

[60] Machado E, Kandzia S, Carilho R, Altevogt P, Conradt HS, Costa J. N-Glycosylation of total cellular glycoproteins from the human ovarian carcinoma SKOV3 cell line and of recombinantly expressed human erythropoietin. Glycobiology. 2011;21(3):376–86. Epub 2010/10/30. doi: 10.1093/glycob/cwq170 . 21030537 10.1093/glycob/cwq170

[61] Beheshti Zavareh R, Sukhai MA, Hurren R, Gronda M, Wang X, Simpson CD, et al Suppression of cancer progression by MGAT1 shRNA knockdown. PLoS One. 2012;7(9):e43721 Epub 2012/09/08. doi: 10.1371/journal.pone.0043721 ; PubMed Central PMCID: PMC3434202. 22957033 10.1371/journal.pone.0043721 PMC3434202

[62] Dennis JW, Laferte S, Waghorne C, Breitman ML, Kerbel RS. Beta 1–6 branching of Asn-linked oligosaccharides is directly associated with metastasis. Science. 1987;236(4801):582–5. Epub 1987/05/01. . 2953071 10.1126/science.2953071

[63] Granovsky M, Fata J, Pawling J, Muller WJ, Khokha R, Dennis JW. Suppression of tumor growth and metastasis in Mgat5-deficient mice. Nat Med. 2000;6(3):306–12. Epub 2000/03/04. doi: 10.1038/73163 . 10700233 10.1038/73163

[64] Nyame AK, Leppanen AM, DeBose-Boyd R, Cummings RD. Mice infected with Schistosoma mansoni generate antibodies to LacdiNAc (GalNAc beta 1—>4GlcNAc) determinants. Glycobiology. 1999;9(10):1029–35. Epub 1999/10/16. . 10521539 10.1093/glycob/9.10.1029

[65] van Remoortere A, Hokke CH, van Dam GJ, van Die I, Deelder AM, van den Eijnden DH. Various stages of schistosoma express Lewis(x), LacdiNAc, GalNAcbeta1-4 (Fucalpha1-3)GlcNAc and GalNAcbeta1-4(Fucalpha1-2Fucalpha1-3)GlcNAc carbohydrate epitopes: detection with monoclonal antibodies that are characterized by enzymatically synthesized neoglycoproteins. Glycobiology. 2000;10(6):601–9. Epub 2000/05/18. . 10814702 10.1093/glycob/10.6.601

