# Supplementary figures and images for: The directed migration of gonadal distal tip cells in Caenorhabditis elegans requires NGAT-1, a ß1,4-N-acetylgalactosaminyltransferase enzyme
Source: PLoS One. 2017 Aug 17;12(8):e0183049. doi: 10.1371/journal.pone.0183049 (PMC5560668; doi:10.1371/journal.pone.0183049)

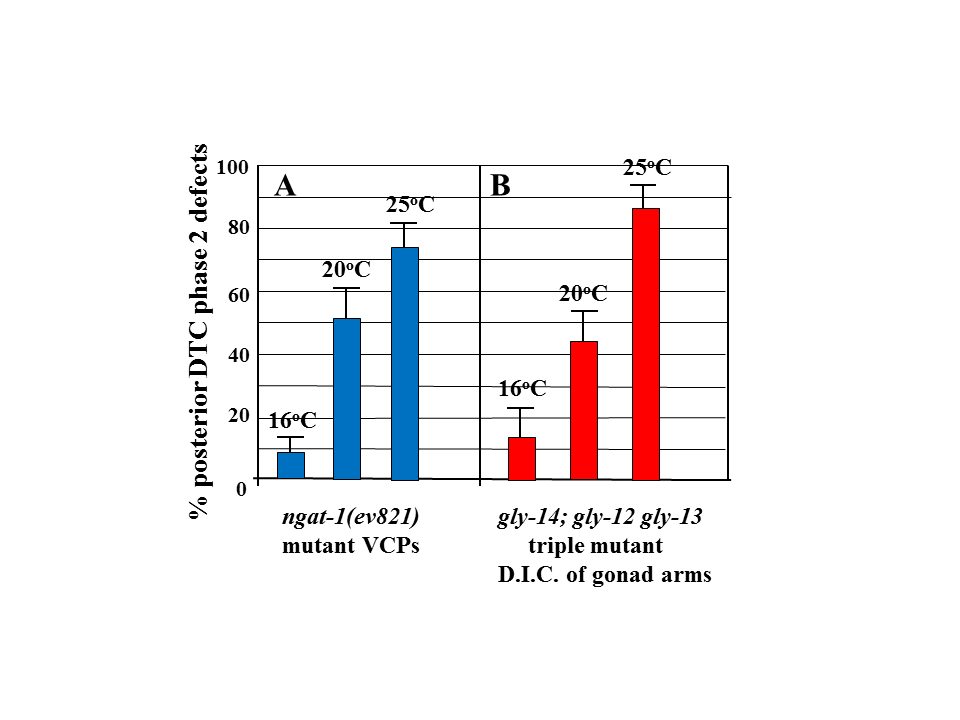

Supplement: S1 Fig — Bars indicate the penetrance of DTC phase 2 errors when grown >2 generations at the indicated temperature. (A) ngat-1(ev821) posterior DTC migration defects were scored as VCPs by stereomicroscopy, whereas, (B) GnT1/gly-14; gly-12 gly-13 mutant DTC defects were scored by D.I.C. microscopy. Error bars represent z-test based 95% confidence intervals for a proportion. (TIF) [file pone.0183049.s001.tif]

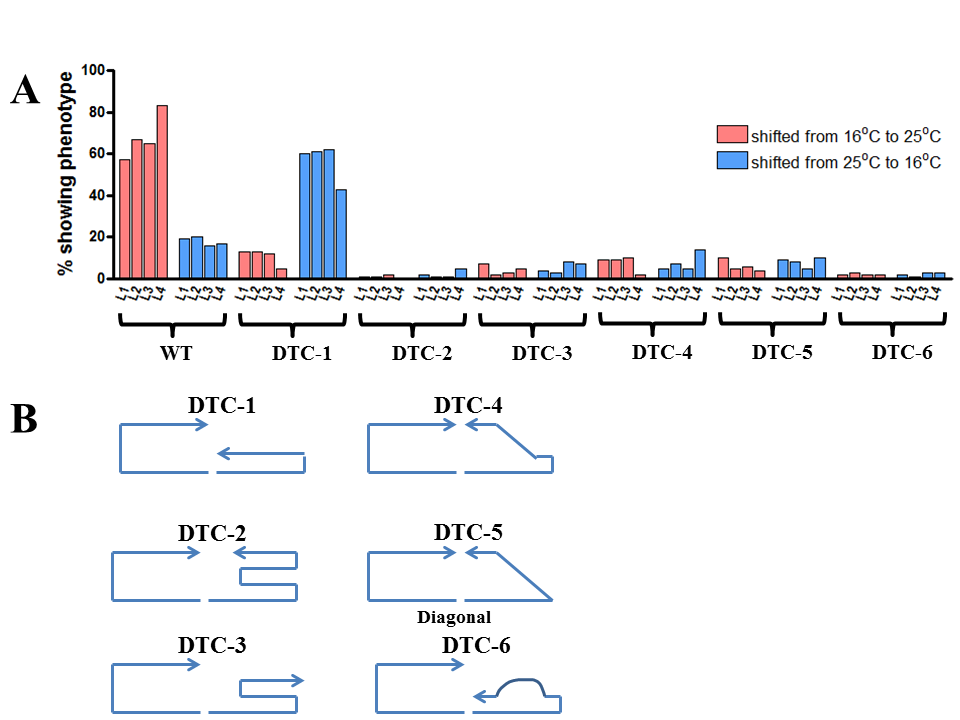

Supplement: S2 Fig — (A) Bars shows the penetrance of phase 2 posterior DTC migration failures of the types (DTC-1 to DTC-6) represented diagrammatically in panel B. L1-L4 = larval stages 1–4. Note that in a different plot of these data, shown in Fig 3, the DTC-5 category is not included as phase 2 errors because many similar trajectories were later found in wild-type animals grown at 25°C. (TIF) [file pone.0183049.s002.tif]

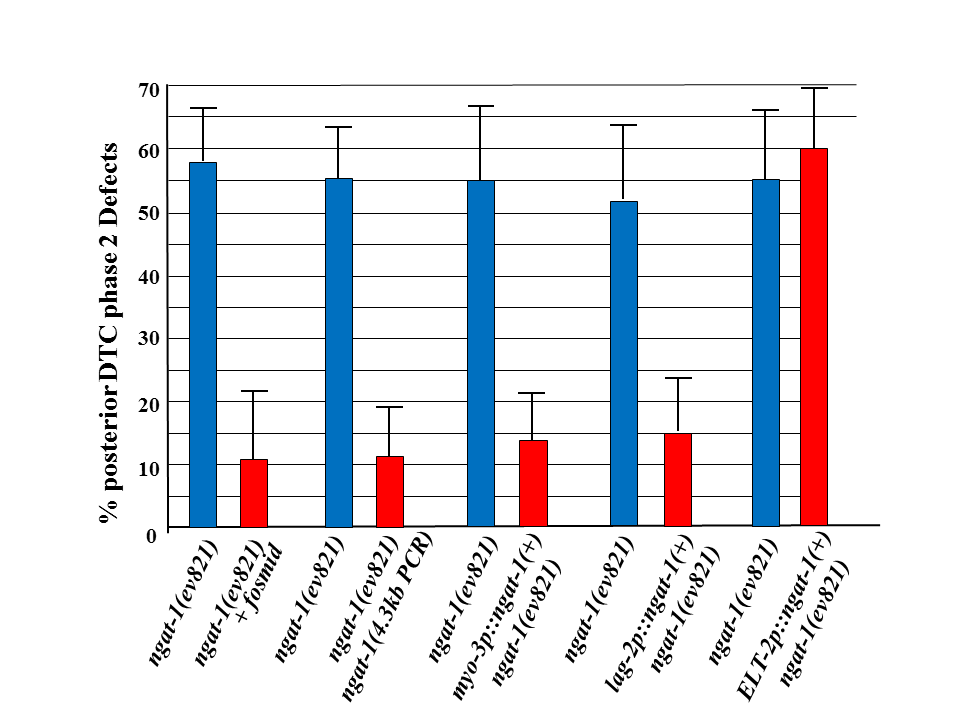

Supplement: S3 Fig — Constructs carrying ngat-1(+) driven by different regulatory regions were made as described in Materials and methods. These include 1) ngat-1(+) in fosmid WRM0637aH09, 2) a 4.3 PCR product encompassing W02B12.11, 3) ngat-1(+) driven by body wall muscle specific promoter myo-3p, 4) DTC-specific promoter lag-2, and intestine-specific promoter elt-2 (see Materials and methods). These results show that W02B12.11/ngat-1(+) expression in body wall muscles or in DTCs (see caveat in Discussion) is largely sufficient to rescue the ngat-1(ev821) mutant DTC migration defects of animals grown at 25°C, whereas, expression in embryonic intestine by the elt-2 promoter does not rescue these defects. Error bars represent z-test based 95% confidence intervals for a proportion. (TIF) [file pone.0183049.s003.tif]

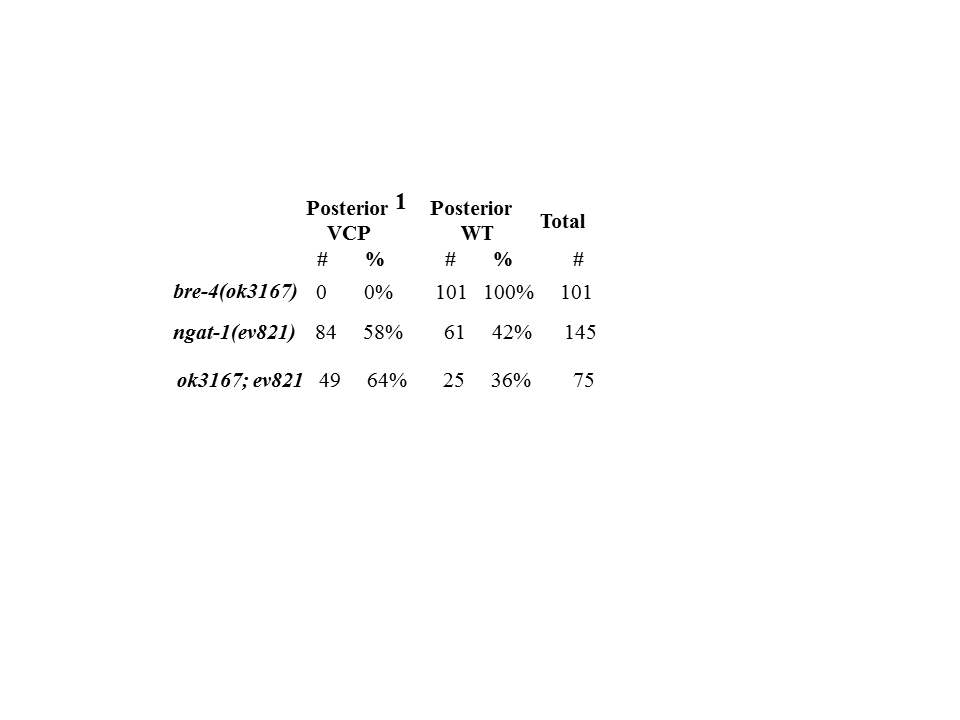

Supplement: S1 Table — 1An ngat-1(ev821); bre-4(ok3167) double mutant—constructed by standard methods—was grown in parallel with both single mutants for two generations at 23°C before monitoring VCPs in L4 animals. Shown are number (#) of gonad arms scored and percentage (%) of animals with given VCP phenotype. (TIF) [file pone.0183049.s004.tif]

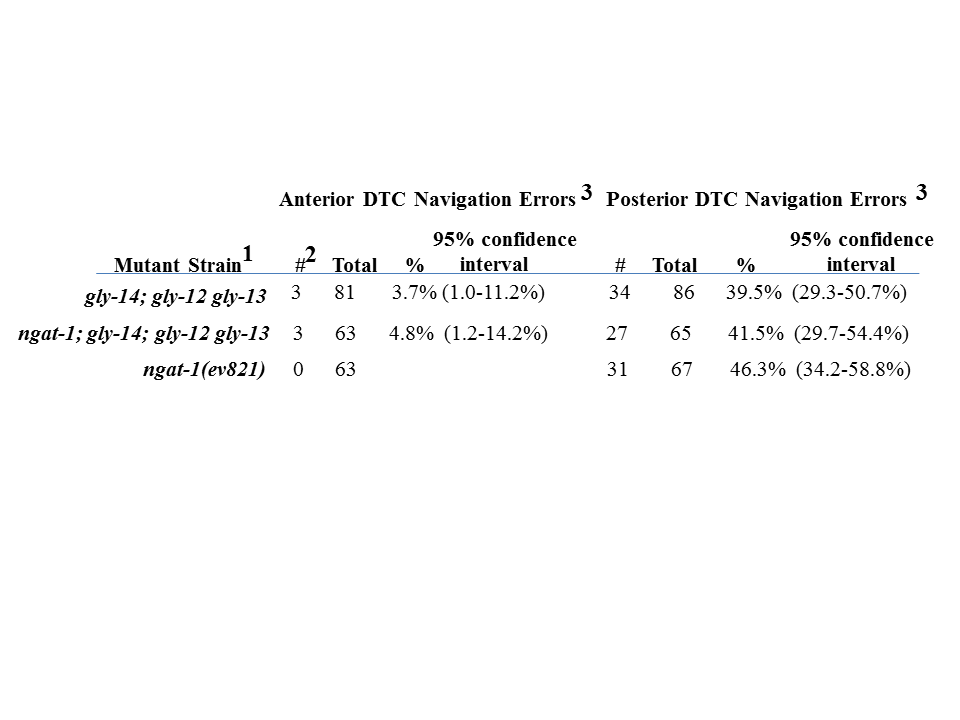

Supplement: S2 Table — 1Number (#) and percentage (%) of animals with phase 2 posterior DTC migration defects. 2Two of the 3 anterior DTC navigation errors (in the triple and quadruple mutants) were premature phase 1 migration toward the dorsal side and 1 was a phase 2 error. 3One anterior DTC migrated along a normal 3 phase trajectory except on the left instead of the right side epidermis in the triple mutant. There was 1, 1 and 3 otherwise normally guided but wrong-sided posterior DTC migrations in the triple, quadruple, and single mutant, respectively. (TIF) [file pone.0183049.s005.tif]

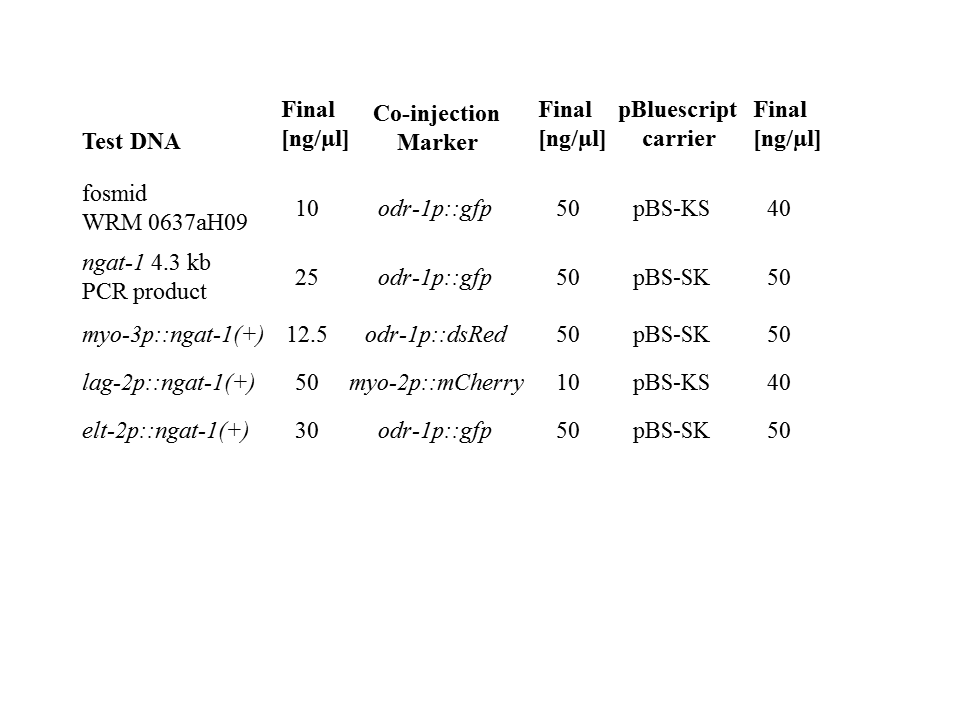

Supplement: S3 Table — (TIF) [file pone.0183049.s006.tif]

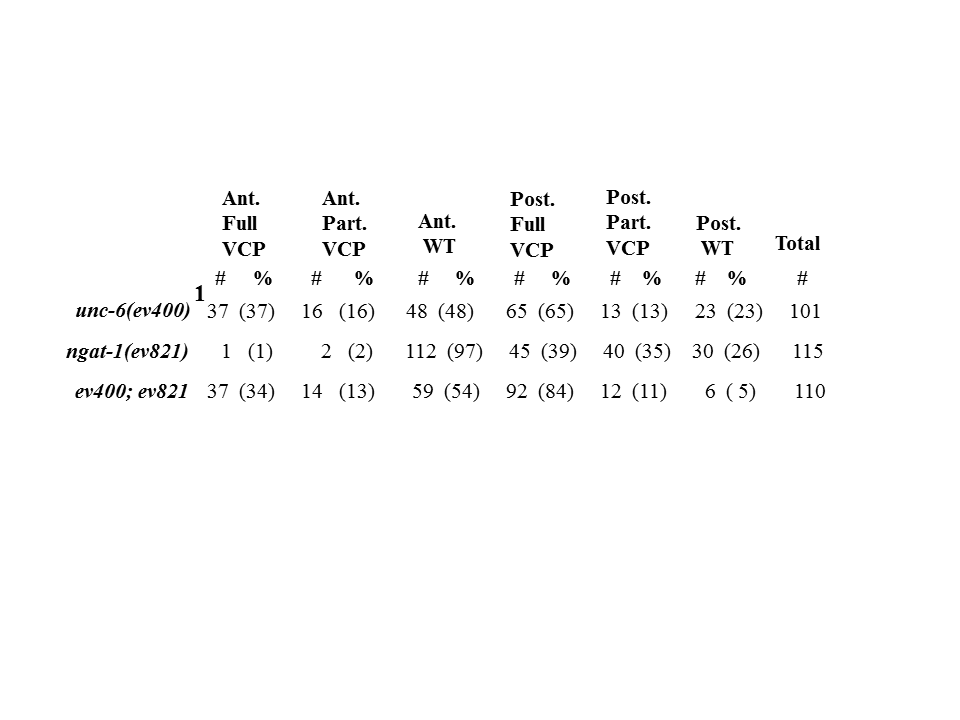

Supplement: S4 Table — 1An ngat-1(ev821); unc-6(ev400) double mutant—constructed by standard methods—was grown in parallel with both single mutants for two generations at 25°C before scoring VCPs in L4 animals. Ant. = Anterior DTC; Post. = Posterior DTC; Full = full and near-full, Part. = Partial; VCP = Ventral Clear Patch, # = number of gonad arms scored. % = percentage of animals with a given VCP phenotype. (TIF) [file pone.0183049.s007.tif]

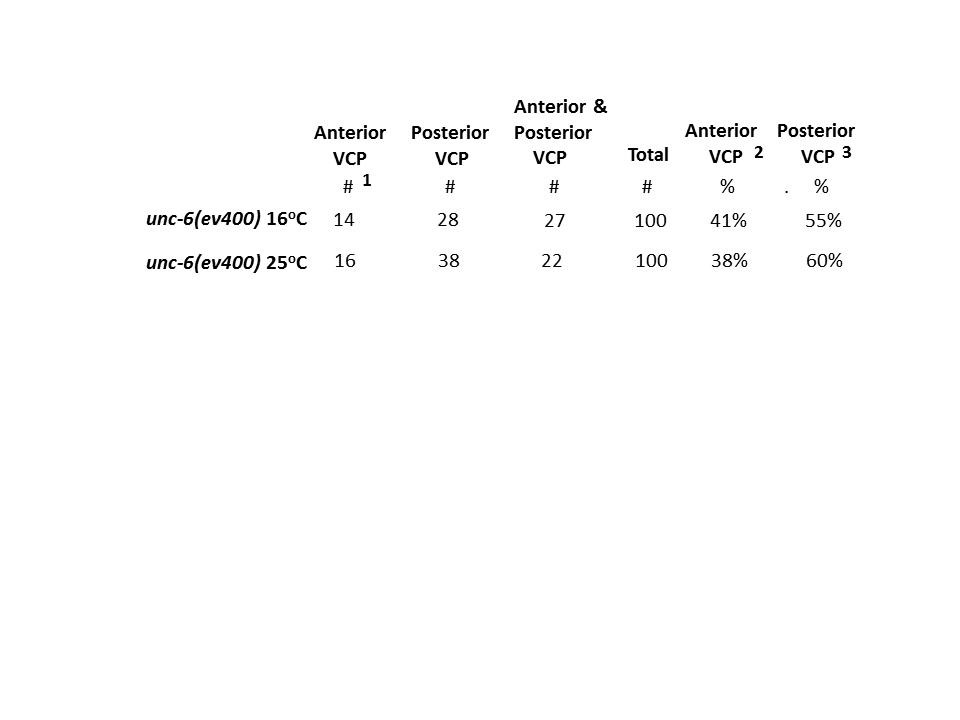

Supplement: S5 Table — 1Number (#) of gonad arms scored following growth for two generations at 16°C or 25°C and percentage (%) of animals with a given VCP phenotype. 2 p = 0.66 by 2-tailed z-test. 3 p = 0.47 by 2-tailed z-test. (TIF) [file pone.0183049.s008.tif]
